# Elastic network modeling of cellular networks unveils sensor and effector genes that control information flow

**DOI:** 10.1371/journal.pcbi.1010181

**Published:** 2022-05-31

**Authors:** Omer Acar, She Zhang, Ivet Bahar, Anne-Ruxandra Carvunis

**Affiliations:** 1 Joint Carnegie Mellon University-University of Pittsburgh Computational Biology PhD Program, University of Pittsburgh, Pittsburgh, Pennsylvania, United States of America; 2 Department of Computational and Systems Biology, University of Pittsburgh School of Medicine, Pittsburgh, Pennsylvania, United States of America; 3 Pittsburgh Center for Evolutionary Biology and Medicine (CEBaM), University of Pittsburgh, Pittsburgh, Pennsylvania, United States of America; Santa Fe Institute, UNITED STATES

## Abstract

The high-level organization of the cell is embedded in indirect relationships that connect distinct cellular processes. Existing computational approaches for detecting indirect relationships between genes typically consist of propagating abstract information through network representations of the cell. However, the selection of genes to serve as the source of propagation is inherently biased by prior knowledge. Here, we sought to derive an unbiased view of the high-level organization of the cell by identifying the genes that propagate and receive information most effectively in the cell, and the indirect relationships between these genes. To this aim, we adapted a perturbation-response scanning strategy initially developed for identifying allosteric interactions within proteins. We deployed this strategy onto an elastic network model of the yeast genetic interaction profile similarity network. This network revealed a superior propensity for information propagation relative to simulated networks with similar topology. Perturbation-response scanning identified the major distributors and receivers of information in the network, named effector and sensor genes, respectively. Effectors formed dense clusters centrally integrated into the network, whereas sensors formed loosely connected antenna-shaped clusters and contained genes with previously characterized involvement in signal transduction. We propose that indirect relationships between effector and sensor clusters represent major paths of information flow between distinct cellular processes. Genetic similarity networks for fission yeast and human displayed similarly strong propensities for information propagation and clusters of effector and sensor genes, suggesting that the global architecture enabling indirect relationships is evolutionarily conserved across species. Our results demonstrate that elastic network modeling of cellular networks constitutes a promising strategy to probe the high-level organization and cooperativity in the cell.

## Author summary

A central goal of systems biology is to understand how biomolecules cooperate through direct and indirect relationships. However, indirect relationships have been notoriously hard to quantify. In protein biophysics, there is a long tradition of quantifying indirect allosteric relationships between amino acids using elastic network models (ENMs). Here, we show that ENMs can be adapted to build global dynamic models of the cell and quantify, unbiasedly for the first time, how much individual biomolecules indirectly influence each other. We illustrate how this approach can be used to infer signaling pathways using budding yeast as a proof of concept. We also show that the global dynamic architecture of genetic networks may have evolved to maintain a high potential for indirect cooperative relationships. In budding yeast, human and fission yeast, we identify specific clusters of genes with key roles in distributing (effectors) or receiving (sensors) biological information through indirect relationships. We propose that elastic network modeling of cellular networks is a promising avenue for tackling current challenges in systems biology.

## Introduction

Cellular networks are abstract representations of the relationships between genes or between their encoded products. Network nodes represent genes or gene products while network edges represent physical (e.g., protein interactions) or functional (e.g., epistatic genetic interactions) relationships between nodes [1]. In-depth analysis of the local connectivity of nodes of interest allows to identify biological modules [2] or disease-associated groups of genes [3] or to elucidate unknown gene functions [4,5]. Global analysis of the interplay between unconnected, seemingly unrelated nodes (“indirect relationships”) can provide a broader understanding of the overarching biological and physical mechanisms that govern cellular systems. For example, the study of indirect relationships helps with predicting how constellations of diverse genetic and environmental signals are sensed to modulate disease risk [6], or with defining how the molecular relationship between distinct cellular processes mediate genotype-phenotype relationships [7]. In sum, it is important to study both direct and indirect relationships between nodes in cellular networks to decipher the biological principles underlying the functional organization of the cell [8].

The study of indirect relationships in cellular networks poses a computational challenge because each network node may be indirectly connected to any other network node through multiple paths, resulting in a complex combinatorial landscape [9]. Network propagation (also referred to as information transfer or geometric diffusion) methods [10–12] have been widely used to identify meaningful indirect relationships between genes [13–15] or within biomolecular structures [16]. The basic principle of these methods is to model a diffusion process, often as a Markovian process, starting from a source node, akin to the flow of a liquid or heat in a solid matter, and to calculate the amount of diffusion from the source node to other nodes in the network. This amount of diffusion across the network is used as a metric quantifying the indirect relationship, with nodes receiving the most information being ranked as most likely to be engaged in a meaningful indirect relationship with the source node. For some applications, this is equivalent to a random walk with restart process on the network nodes [15]. Network propagation methods have been applied to a wide range of biological problems, from identifying disease-related genes [17,18] to protein homology detection [19].

An important caveat for the use of network propagation methods in cellular networks is the requirement for prior information to select source genes, such as disease genes. This introduces an inherent bias that hampers the discovery of novel indirect relationships that may be critical for cellular function but do not involve genes that would be chosen as sources based on prior knowledge. To obtain a comprehensive understanding of a network’s indirect relationships, an unbiased approach is needed in which all nodes would be considered as possible sources and all possible indirect relationships would be investigated. With such an unbiased method, one could possibly identify key propagation-mediating genes without relying on prior knowledge and extract the strongest indirect relationships between them. Indeed, not all genes would be expected to engage equally in indirect relationships. Some genes that are involved in multiple cellular processes might be very effective at propagating signals throughout the cell. Other genes might be involved in sensing and integrating signals from diverse sources in the cell to coordinate cellular responses to environmental changes–a property that cannot currently be predicted based on cellular network connectivity to our knowledge. In sum, novel approaches are needed to illuminate the global architecture of indirect relationships in the cell.

**Fig 1:**
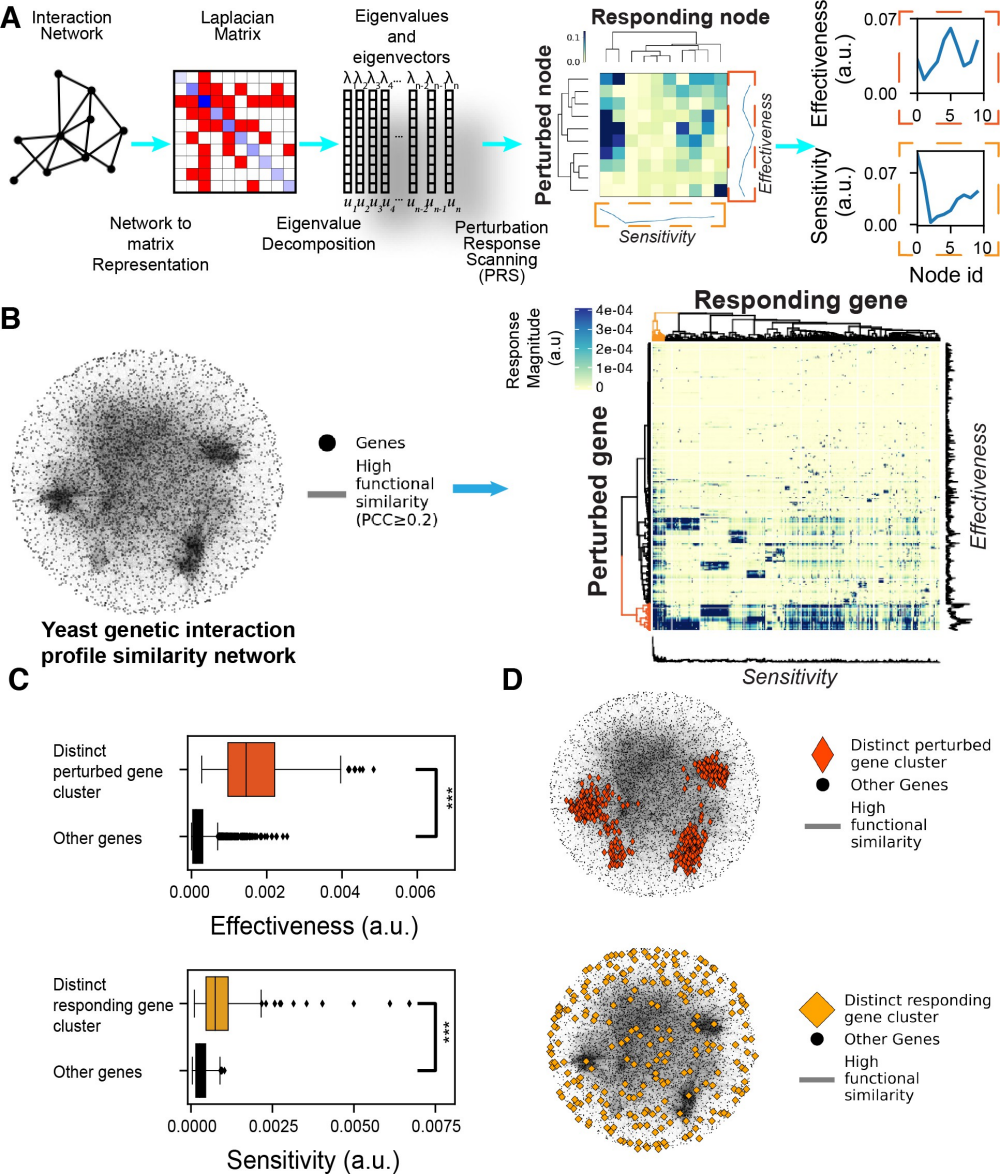
Perturbation Response Scanning (PRS) applied to the yeast genetic interaction profile similarity network (GI PSN). A) The PRS strategy illustrated on a toy network. The network is first transformed into a Laplacian matrix describing the network connectivity. The Laplacian matrix shows the degree of each node on the diagonal (shades of *blue*, darker is the higher degree) and connectivity of each node on the non-diagonal (*red*). Eigenvalue decomposition of the Laplacian yields the eigenvalues and eigenvectors used to calculate the covariance matrix. Each row of the covariance matrix is then normalized by the diagonal element resulting in an asymmetric PRS matrix where each row corresponds to a perturbed node, each column corresponds to a responding node and the colors show the magnitude of the response in arbitrary units (a.u., also used for the remaining figures). Row and column averages of the PRS matrix represent the effectiveness (*right ordinate*) and sensitivity (*lower abscissa*) of each node, respectively. B) PRS analysis of the GI PSN (*left*) yields the PRS matrix shown on the *right*. The nodes on the network (*black dots*) are the genes and the edges (*gray lines*) represent high profile similarity (Pearson’s correlation coefficient of pairs of genetic interaction profiles (PCC) ≥ 0.2). Gray shaded regions in the center of the network are the result of high connectivity and number of edges in the center. This network representation is used throughout the paper. *Colored parts* on the dendrograms along the two axes of the PRS matrix indicate distinct perturbed and responding gene clusters that are separated from the rest of the genes at the top level of the dendrograms (*red* and *orange*, respectively). These colors are used in the panels C and D to show the properties of the genes in these two clusters. C) Effectiveness (*top*) and sensitivity (*bottom*) boxplots showing the differences between perturbed and responding gene clusters, respectively (***: *p* < 0.001, permutation test). D) Representation of the distinct perturbed gene (*top*) and responding gene (*bottom*) clusters within the GI PSN.

To achieve this goal, we leveraged a perturbation-response scanning (PRS) strategy initially developed for the unbiased identification of allosteric interactions within biomolecular structures [20–22]. For PRS analysis, the structures (proteins, their complexes or assemblies, or chromosomes) are represented by elastic network models (ENMs) [23] where each network node represents a physical entity (e.g., a residue, domain, monomer, or gene locus in the chromatin) [24] and each network edge is modeled as a spring that represents a physical interaction between the nodes. ENM representation allows for the application of forces perturbing every node in the network, generating a perturbation effect that propagates throughout the network and enables quantitative measurement of the cooperative motions/responses of all other nodes (Fig 1A). These measurements derive directly from the Laplacian matrix deduced from the network topology (Fig 1A). Therefore, PRS provides a unique mathematically exact analytical solution for (i) the strength of cross-correlations between the fluctuations of each pair of network nodes (indirect relationships) and (ii) the ability of each node to transmit perturbation effects to other nodes (“effectiveness”) and to receive those effects transmitted by other nodes (“sensitivity”), through indirect relationships [21].

We reasoned that PRS could be successfully extended from molecular structures to cellular networks. Indeed, the connection between PRS and graph-theory-based analyses is well established. For example, the central component of the PRS analysis—the covariance matrix deduced from the ENMs—is directly proportional to the commute times predicted by Markovian propagation process to measure the efficiency of information transduction between pairs of nodes in a network [16]. Furthermore, spring-based modeling has already proven to be valuable for both visualization [25] and functional inference [26] in cellular networks. Therefore, the PRS strategy has the potential to enable systematic and unbiased identification of critical propagation-mediating nodes and to derive a global, unbiased view of indirect relationships in cellular networks.

Here, we adapted the PRS strategy to identify the key propagation-mediating genes and the indirect relationships between these genes in the comprehensive genetic interaction profile similarity network (GI PSN) generated for *S*. *cerevisiae* by Costanzo *et al*. [27]. In this network, genes are linked when their respective deletions impart similar effects on cell growth in the context of deletions of nearly all other genes in the genome one by one [28]. It is now well-established that genes with direct interactions in the GI PSN tend to share similar molecular functions, and edges in the GI PSN are commonly interpreted as indicating high “functional similarity” [29]. In addition, modules of tightly connected genes in the GI PSN often correspond to molecular machines or pathways [27], which are themselves integrated into increasingly larger subnetworks representing the hierarchical organization of cellular processes [30,31]. Indirect relationships in the GI PSN, therefore, represent the functional relationships between distinct cellular pathways and processes.

## Results

### PRS clusters genes based on their potential to receive and transmit information

The GI PSN generated by Costanzo et al. [27] contains 5,183 nodes (genes) and 39,816 edges (functional similarity, defined as pairwise correlations of genetic interaction profiles with PCC ≥ 0.2). We constructed an ENM representation of the GI PSN and applied the PRS strategy to this network by perturbing each gene individually and measuring the responses of the other genes. This resulted in a 5,183-by-5,183 PRS matrix (Fig 1B) representing the perturbation-response relationship between all pairs of genes. Hierarchical clustering of the PRS matrix rows and columns delineated groups of genes based on their information propagation profiles. Notably, one cluster of perturbed genes and one cluster of responding genes were distinctly separated from the rest of the genes in the dendrograms (Fig 1B, colored clusters on the dendrograms). The genes in these two distinct clusters displayed higher effectiveness and sensitivity, respectively, than the rest of the genes in the network (Fig 1C, *p* <0.001, permutation test). Next, we mapped the genes belonging to these distinct clusters on the network. The distinct cluster of perturbed genes corresponded to highly connected, central regions of the network. In contrast, the members of the distinct cluster of responding genes were distributed throughout the network, with a tendency to be located in peripheral locations (Fig 1D). Overall, PRS-based clustering identified two classes of genes; one with high effectiveness localized in densely connected regions of the network; and another with high sensitivity distributed at loosely connected regions of the network.

### GI PSN displays a remarkable propensity for indirect relationships

**Fig 2:**
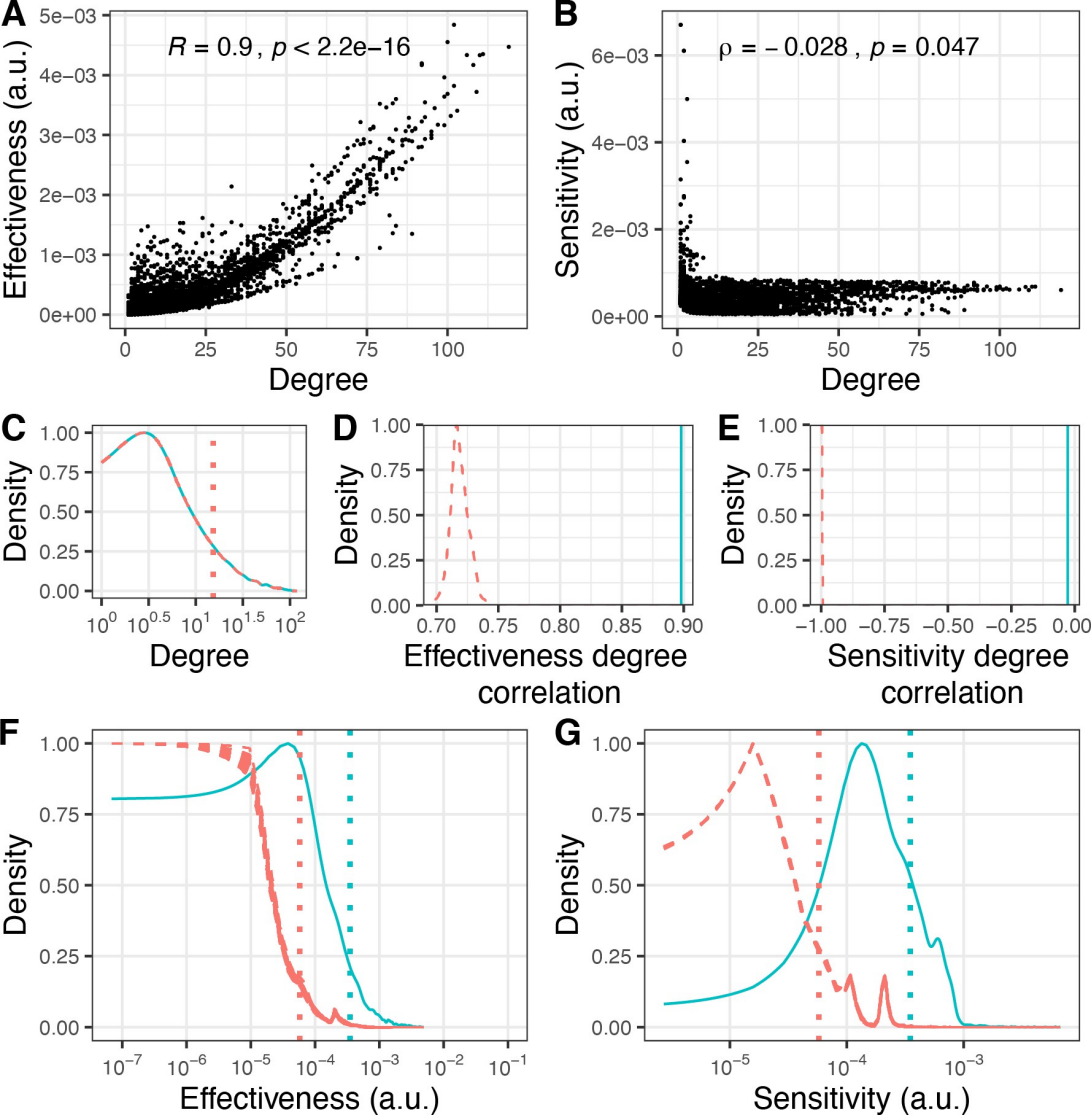
The GI PSN displays superior propagation potential compared to randomly rewired networks with identical degree distributions. A) Degree and effectiveness scatter plot shows strong correlation between degree and effectiveness in the GI PSN (Pearson’s correlation coefficient, *R* = 0.9). B) Degree and sensitivity scatter plot shows no correlation between degree and sensitivity in the GI PSN (Spearman’s rank correlation, *ρ* = −0.028). C) Degree distributions for the GI PSN (*cyan*) and 100 rewired networks (*red*). These distributions overlap by design. D) The correlation between degree and effectiveness is significantly higher in the GI PSN (*cyan vertical line*) than that expected for the rewired networks (*dashed red distribution*, average *R* = 0.72, *p* < 0.01, empirical *p*-value). E) The correlation between degree and sensitivity is significantly weaker in the GI PSN (*cyan vertical line*) than expected from rewired networks (*dashed red distribution*, average *ρ* = −0.99, *p* < 0.01, empirical *p*-value). Nodes in the GI PSN (*cyan distributions*) exhibit significantly higher effectiveness (F) and sensitivity (G) compared to random network nodes (*red dashed distributions*, *p* < 0.01, empirical *p*-value).

The local connectivity of each node can be described by its degree (number of neighbors), and the behavior of a network is largely characterized by its degree distribution [32]. Thus, we sought to understand how the degree of nodes and the degree distribution of the GI PSN influence effectiveness and sensitivity profiles. We found that effectiveness was highly correlated with degree (Fig 2A, *R* = 0.9). In contrast, sensitivity was not (*ρ* = −0.028) although nodes with low degrees (degree < 10) tended to show higher sensitivity (Fig 2B, *ρ* = −0.52 for degree < 10). To investigate the significance of these results, we compared the GI PSN to 100 random networks generated by rewiring the GI PSN edges while keeping the degree distribution constant (Fig 2C). The GI PSN displayed a significantly stronger degree-effectiveness correlation and a significantly weaker degree-sensitivity correlation than rewired networks (Fig 2D and 2E, *p* < 0.01, empirical *p*-value). Thus, the topology of the GI PSN enables perturbation effects to propagate in a manner that cannot be explained simply by its degree-related characteristics.

Next, we compared the effectiveness and sensitivity distributions of the GI PSN and the rewired networks. The shapes of effectiveness and sensitivity distributions bear some interesting resemblance between the real and the rewired networks (Fig 2F and 2G, red dotted and cyan solid curves), but nodes in the GI PSN have overall higher effectiveness and sensitivity values than nodes in the random networks (Fig 2F and 2G, *p* < 0.01, empirical *p*-value). This finding was robust to variations in the stringency of functional similarity chosen to define the GI PSN edges: networks constructed using different PCC thresholds consistently displayed stronger effectiveness and sensitivity profiles compared to their rewired equivalents (S1 Fig). Interestingly, the difference in the distributions derived from real and rewired networks was always greater in the case of sensitivity than effectiveness. This is likely due to the lesser dependence on the degree observed for sensitivity relative to effectiveness (Fig 2A).

Overall, since effectiveness and sensitivity measure the potential of the nodes to transmit and receive information through indirect relationships, our results indicate that the GI PSN harbors a remarkable propensity for indirect relationships in general, and for sensitivity especially, relative to expectations based on degree-related characteristics alone. This propensity originates from the overall network topology of the GI PSN, accounted for by the entire Laplacian matrix—not just the diagonal terms that represent the degree of the individual nodes, but also the off-diagonal terms that define the specific node-node couplings.

### Sensors form “antenna-shaped” biological clusters loosely connected with the GI PSN

**Fig 3:**
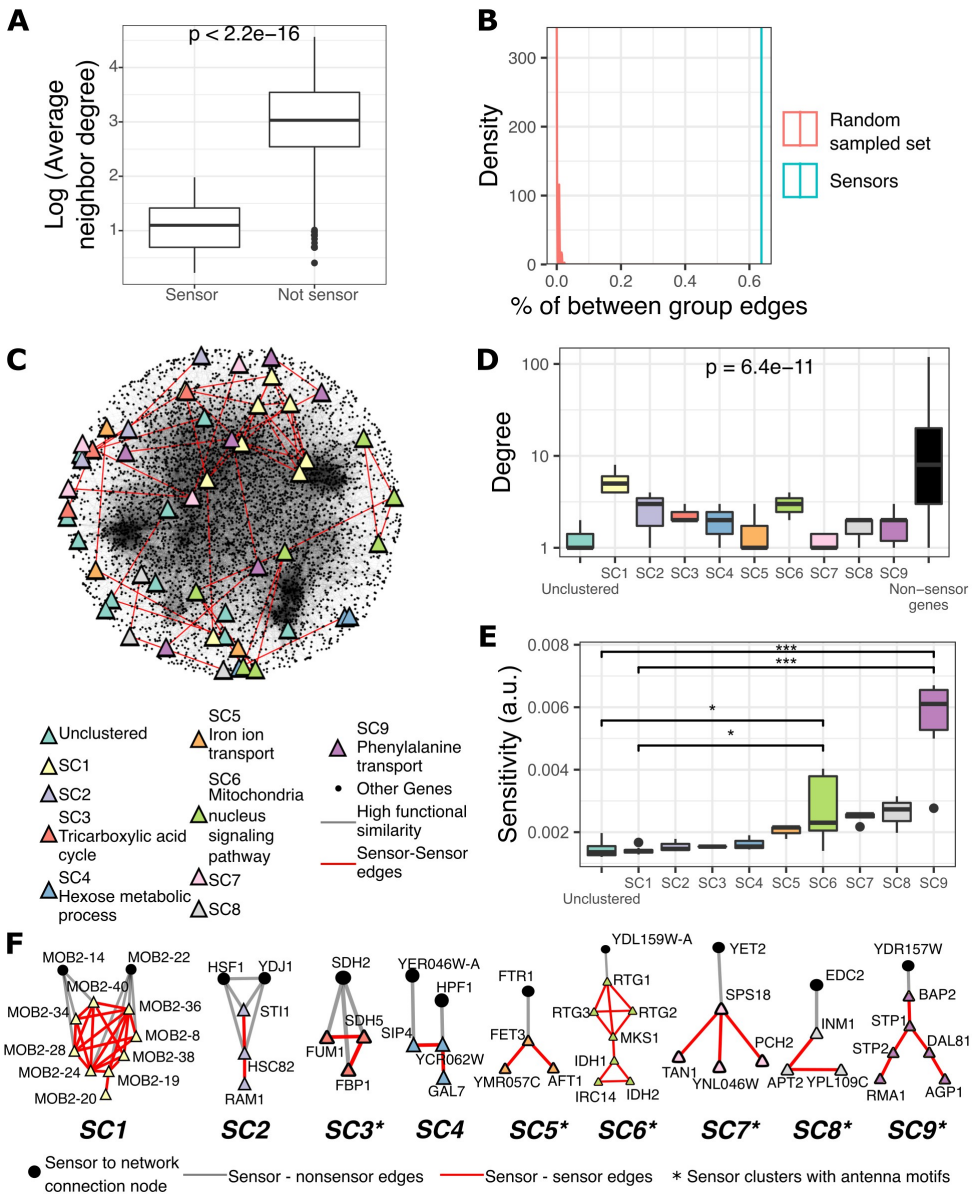
Sensors form biologically coherent low-degree gene clusters on the network periphery. A) The first neighbors of sensors have low average degree relative to the neighbors of other genes in the network (*p* < 0.0001, Wilcoxon signed-rank test). B) Sensors are more densely connected to each other than expected from randomly sampled nodes with the same degree, as measured by the percentage of the between-group (sensor-to-sensor) edges among the total edges the nodes have (sensor group: *cyan vertical line*; groups of randomly sampled nodes with same degree: *red distribution*, ~224 fold, *p* < 0.0001, permutation test with the same degree sampling). C) Representation of sensor genes in the GI PSN. Node colors represent sensor clusters (groups of 3 or more directly connected sensors) and GO enriched clusters are specified in the legend (resampling-based false discovery rate (FDR) < 0.1). Node colors and shapes, and edge colors were used similarly for the panels D, E, F, and the Fig 5. D) Sensor clusters (SCs 1–9) exhibit comparable degrees to each other and significantly lower degree than non-sensor genes in the network (*black* box, *p* < 0.001, Kruskal-Wallis, group-wise comparison including non-sensor genes). E) The sensor clusters have similar sensitivity, except for SC9 (‘phenylalanine transport’) which shows the highest sensitivity (*: *p* < 0.1, ***: *p* < 0.001 for corrected *p*-values calculated by Mann-Whitney). F) Topology of sensor clusters and the nodes that connect these clusters to the rest of the network are shown (*Triangles*: sensors, *Circles*: non-sensor connecting node, *asterisk*: clusters with antenna motifs).

Genes with higher sensitivity are more likely to be involved in indirect relationships due to their ability to integrate information from other parts of the network. We defined genes with the highest sensitivity (top 1%) as sensor genes (*n* = 52). Sensors tended to have low degrees and, in many cases, had only a single connection (Fig 2B). In protein structure networks, sensor residues identified by PRS tend to be connected to residues with relatively higher effectiveness [21]. However, this was not the case in the GI PSN where sensor genes tended to be connected to other low-degree genes (Fig 3A, *p* < 0.0001, Wilcoxon signed-rank test) with low effectiveness (Fig 2A). In fact, the first neighbors of sensors had degrees about six orders of magnitude smaller than the first neighbors of non-sensor genes (Fig 3A). To understand what distinguishes sensors from the many other low-degree genes in the network that did not display high sensitivity, we studied their topologies and biological properties in depth.

We first investigated whether sensors tended to be connected to each other in the GI PSN, which would indicate that they share functional similarities. To this aim, we randomly sampled genes with the same degree as the sensors and calculated the number of the between-group edges relative to the total number of sensor edges. We found that sensors had a strong tendency to connect directly to each other relative to expectations based on the degree-controlled samples (Fig 3B, ~224 fold, *p* < 0.0001, permutation test with the same degree sampling), revealing the existence of sensor clusters. We divided sensors into subnetworks consisting of only sensor genes. We considered subnetworks with at least three nodes as a ‘sensor cluster’ and observed that 41 of 52 sensors in the network formed nine distinct clusters (SC1-9; Fig 3C). Such sensor clusters were not identified when PRS was applied to rewired networks with the same size and same degree distribution as the GI PSN (only 14% of rewired networks had at least one cluster with more than three sensors, S2A Fig). Hence, clusters of low-degree sensor genes are unexpected, and likely represent a heretofore undiscovered biological property of the GI PSN.

Since edges in the GI PSN represent functional similarity, we hypothesized that sensor clusters may correspond to biologically coherent groups of genes. GO enrichment analysis of individual sensor clusters revealed significant associations with specific but distinct biological processes for five of the nine sensor clusters: tricarboxylic acid cycle (TCA) cycle (SC3, resampling-based false discovery rate (FDR) = 0.02), hexose metabolic process (SC4, resampling-based FDR = 0.03), mitochondria-nucleus signaling (SC5, resampling-based FDR = 0.002), iron ion transport (SC6, resampling-based FDR = 0.03) and phenylalanine transport (SC9, resampling-based FDR = 0.006) (Fig 3C, **S1 Data**). In contrast, only 3% of rewired networks showed at least one sensor cluster with a GO enrichment (S2B Fig). While sensor genes had a lower degree than other genes in the network (Fig 3D, *p* < 0.001, Kruskal-Wallis, group-wise comparison including non-sensor genes), we did not observe significant differences in the average node degree between the nine sensor clusters (Fig 3D, *p* = 0.16, Kruskal-Wallis, group-wise comparison excluding non-sensor genes). Intriguingly, the sensor cluster SC9 related to ‘phenylalanine transport’ displayed the highest sensitivity (Fig 3E). Altogether, these analyses show that PRS identified well-defined clusters of sensor genes, each involved in distinct cellular processes, as the nodes most likely to sense signals or integrate information from the rest of the cell through indirect relationships.

We therefore investigated whether sensors and sensor clusters may be involved in cellular signaling. We explored this hypothesis by examining the literature describing previous knowledge about the sensor genes and gene ontology (GO) functional annotations. Generally, the GO enrichments of five of the nine clusters (Fig 3C) indicated roles in sensing, responding to metabolic cues, and cellular transport. Only the SC6, but none of the other eight sensor clusters, mapped to a well-characterized signaling hub (SC6 contained four mitochondria-nucleus signaling genes, *RTG1*, *RTG2*, *RTG3*, and *MKS1*, out of seven sensor genes in the cluster) [33]. Yet, five of the nine sensor clusters (SC4, SC5, SC6, SC7, SC8, and SC9) contained at least one sensor gene annotated with a known signaling function (GO:0023052; seven sensor genes total) or with known functions related to cell communication (GO:0007165; 11 sensor genes total). For example, the SC5 members *AFT1* and *FET3* are annotated with ‘cell communication’ due to their involvement in iron sensing [34]. Further examination showed that additional sensor genes have been described as the targets of signaling pathways. For example, four of six genes in SC9 (*STP1*, *STP2*, *AGP1*, and *BAP2*) are known to be regulated by the Ssy1p–Ptr3p–Ssy5p (SPS) complex, which senses extracellular amino acids [35]. Altogether our analyses revealed that 15 / 52 sensor genes encode known signaling proteins or signaling targets and that 5 / 9 sensor clusters contain at least one gene encoding a known signaling protein or signaling target, although as a group the 52 sensors genes were not enriched in signaling or related gene ontology (GO) terms (resampling-based FDR > 0.1, **S2 Data**). These results suggest that the sensors identified by PRS may correspond to key recipients of cellular signals and that future studies are likely to unveil novel roles in signaling for many of the 37 sensor genes that are not currently known to be involved in signaling.

To understand why these 52 genes and nine clusters, but not other low-degree genes or signaling-related clusters, were identified by PRS as the strongest sensors of cellular information, we inspected their local topology more closely. Interestingly, we found that six of nine sensor clusters were connected to the rest of the network by a single non-sensor node, creating antenna-shaped motifs (Fig 3F). These antenna motifs appeared to form an information bottleneck where the perturbation effects can enter the sensor cluster but cannot escape easily and transfer to other nodes outside of the cluster. We found that similar antenna-shaped motifs also characterized sensor clusters detected by PRS in networks constructed with more stringent cutoffs for functional similarity, even though the number and identity of these clusters were different from those in the GI PSN (S3 Fig). Additionally, the few sensor clusters identified in rewired networks with the same degree distribution tended to show similar antenna motifs (S2C Fig). Thus, the sensitivity of lower degree nodes within antenna motifs, as opposed to those outside the motifs, may be increased by the local accumulation of perturbation effects.

### Effectors form biological clusters centrally integrated within the GI PSN

**Fig 4:**
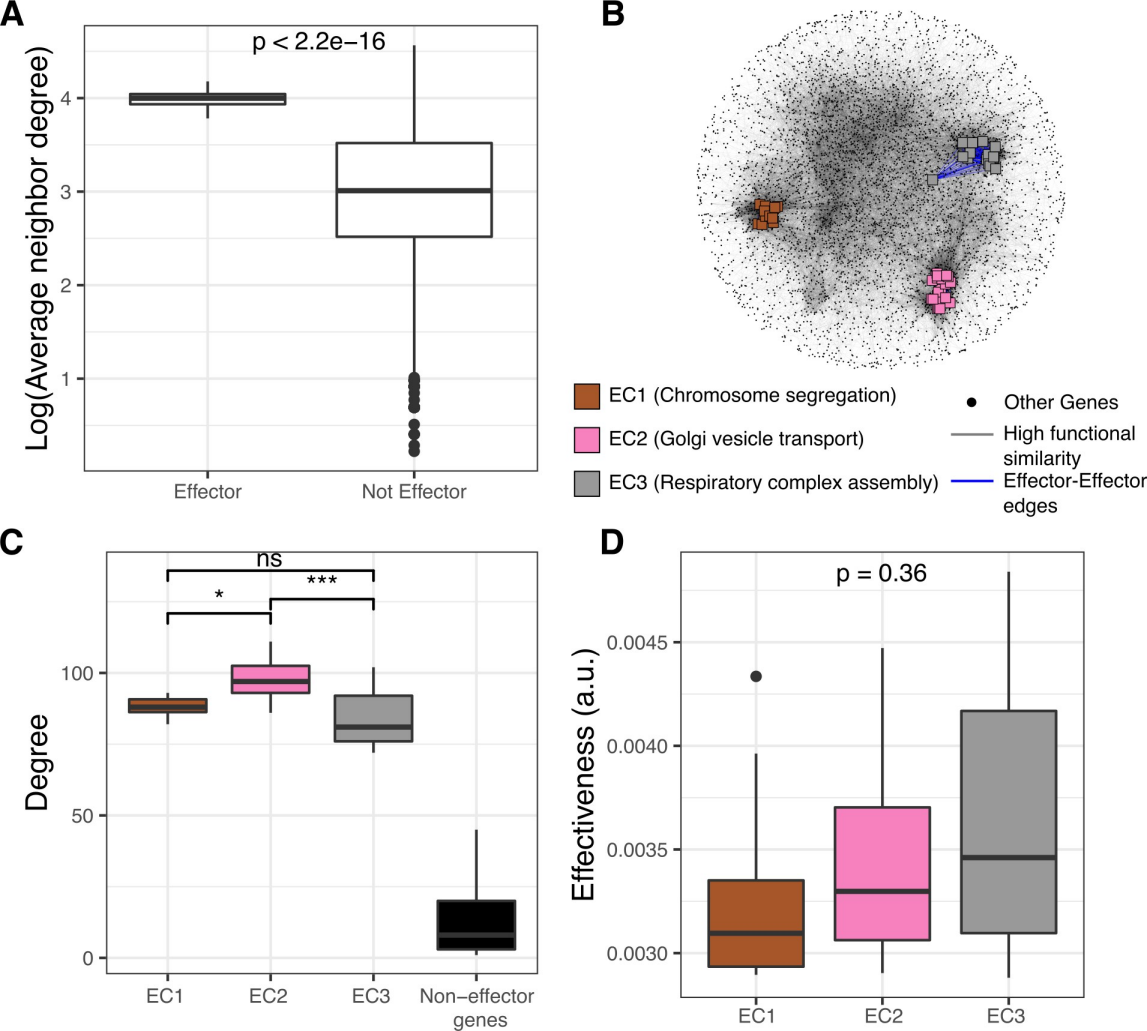
Effectors form biologically coherent high-degree gene clusters at the center of the network. A) The neighbors of effectors have high average degree relative to the neighbors of other genes in the network (*p* < 0.0001, Wilcoxon signed-rank test). B) Representation of effector genes in the GI PSN. Node colors represent effector clusters (ECs) with distinct GO term enrichments (node colors and shapes, and edge colors were used similarly for the following figure panels, resampling-based FDR < 0.1). C) Effectors have a higher degree than other genes and the EC2 with ‘Golgi vesicle transport’ enrichment has a higher average degree than other effector clusters (ns: non-significant, *: *p* < 0.05, ***: *p* < 0.001, for corrected *p*-values calculated by Mann-Whitney). D) Effectiveness values are not significantly different between different clusters of effectors (*p* = 0.36, Kruskal-Wallis).

To investigate the genes that exert the strongest influence on the other genes in the network through indirect relationships, we defined genes with the highest effectiveness (top 1%) as effector genes (*n* = 52) and studied their topological and biological properties. Unlike sensors, effectors tended to connect to other high-degree genes (Fig 4A, *p* < 0.0001, Wilcoxon signed-rank test). However, effector-effector edges consisted of only 7% of all edges involving effectors due to their extremely high degree. Nevertheless, they formed distinct effector clusters (ECs), with no direct connections across different effector clusters. We could separate all 52 effectors into three connected components, each associated with a distinct cellular process based on GO enrichment analysis: chromosome segregation (EC1, resampling-based FDR = 0.002), Golgi vesicle transport (EC2, resampling-based FDR = 0.002), and respiratory complex assembly (EC3, resampling-based FDR = 0.002). Similar effector genes (S4A Fig) and GO term enrichments (S4B Fig) were identified when PRS was applied to networks constructed with more stringent functional similarity thresholds.

We investigated whether the effector genes and effector clusters were involved in biological signaling. EC1 did map to a well-characterized signaling hub with significant GO enrichments for cell cycle checkpoint signaling (GO:0000075, resampling-based FDR = 0.002) and intracellular signal transduction (GO:0035556, resampling-based FDR = 0.048). EC1 was also enriched in chromosome segregation and cell cycle (resampling-based FDR = 0.002 for both). On the other hand, EC2 and EC3 had GO enrichments in Golgi vesicle transport and respiratory complex assembly (and related categories, Fig 4B, resampling-based FDR = 0.002, **S1 Data**) with no explicit relationship to signal transduction. We found that all three clusters display significantly higher average degrees than other genes in the network (Fig 4C, *p* < 0.001, Kruskal-Wallis, group-wise comparison including non-effector genes) and that effectors involved in Golgi vesicle transport have a slightly but significantly higher average degree than effectors from the other two effector clusters (Fig 4C, *p* < 0.001, Kruskal-Wallis, group-wise comparison excluding non-effector genes). However, there was no significant difference in effectiveness values between the three clusters (Fig 4D, *p* = 0.36, Kruskal-Wallis).

In summary, effectors formed three biological clusters that are centrally integrated within the GI PSN while being clearly distinct from each other. While one of the effector clusters, EC1, mapped to a well-characterized signaling hub, the other two mapped to two essential functions, vesicular transport of proteins through the Golgi apparatus and respiratory complex assembly, both of which are highly regulated processes that may require the involvement of highly effective propagation of signals. For example, the trafficking and transport function of the Golgi apparatus is intimately related to multiple intracellular signaling pathways [36,37]; likewise, multiple pathways regulate the mitochondrial respiratory complex assembly [38]. Future analyses might therefore reveal the potential of the genes in EC2 and EC3 to play roles in cellular trafficking, communication and signaling that are currently under-appreciated.

### Systematic detection of indirect relationships in the GI PSN

**Fig 5:**
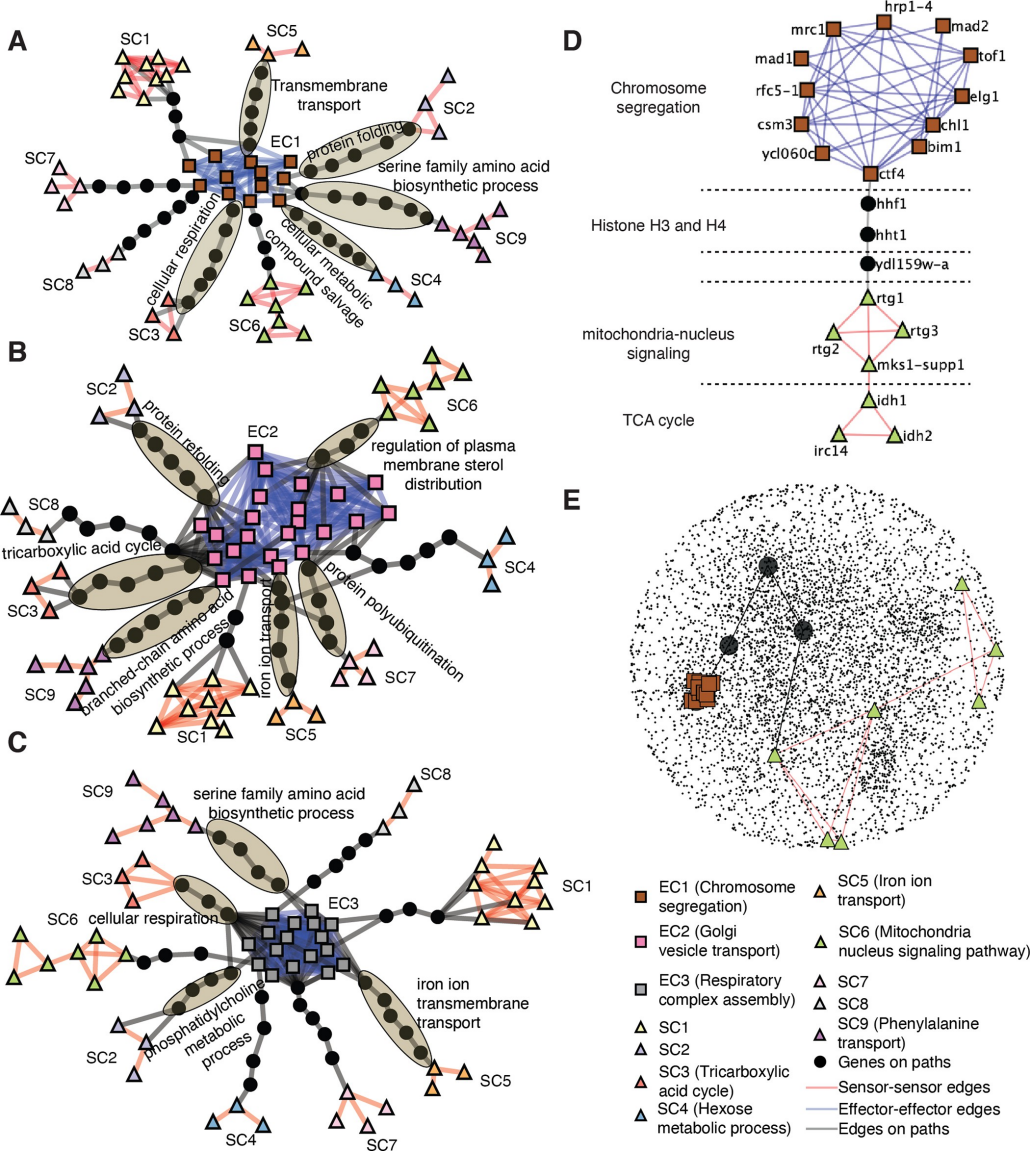
PRS identifies biologically meaningful indirect relationships without relying on prior knowledge. A-C) Connection effector clusters (EC1 to EC3, respectively) to sensor clusters chosen based on shortest paths with the highest propagated information. Paths with GO enrichments are highlighted with ellipses and the most significant GO terms are indicated (resampling-based FDR < 0.1). The identity of all genes in these subnetworks is shown in S5 Fig. D) The shortest path from effector genes involved in chromosome segregation (EC1) to sensors involved in mitochondria-nucleus signaling (SC6), as identified by shortest path between the effector gene *CTF4* and the sensor gene *RTG1*. E) Representation of the path shown in (D) within the GI PSN.

Our PRS analyses identified effector and sensor genes as the key propagation-mediating genes that control information flow in the GI PSN. To investigate the indirect relationships between effectors and sensors, we extracted the shortest paths connecting the three effector clusters to the nine sensor clusters. We first identified 4,815 shortest paths between effector and sensor genes, including multiple shortest paths between individual effector-sensor cluster pairs (S5A Fig). We selected the 27 paths with the highest total perturbation effects per effector-sensor cluster pair as representing the strongest and possibly most meaningful indirect relationships between effectors and sensors (Figs 5A–5C and S5B–S5D). Most (15 of 27) of these paths comprised biologically coherent groups of genes as shown by GO enrichment analysis (Fig 5A–5C; resampling-based FDR < 0.1).

Based on the PRS theory, the indirect relationships between effector and sensor clusters are likely to constitute the pillars of the higher-order organization of the GI PSN. The paths identified here therefore represent novel biological hypotheses for unraveling the molecular mechanisms of intra-cellular communications between distinct cellular processes. To gain a better understanding of the predictive value of PRS paths between effector and sensor clusters, we further inspected the path between the cluster of effectors related to chromosome segregation (EC1) and the cluster of sensors related to mitochondria-nucleus signaling (SC6, Fig 5D and 5E). The nodes on this path showed no biological enrichments based on prior knowledge (resampling-based FDR > 0.1, Fig 5A).

The sensor cluster SC6 consisted of two smaller sub-clusters corresponding to distinct but related cellular pathways. The first sub-cluster contained the *RTG1*, *RTG2*, *RTG3*, and *MKS1* genes, which control mitochondria-nucleus signaling, also known as the retrograde (RTG) pathway. This pathway controls the production of nuclear-encoded mitochondrial genes [33]. Rtg1p, Rtg2p, Rtg3p are positive regulators of the RTG pathway, while Mks1p is a negative regulator [39,40]. The second sub-cluster consisted of *IDH1*, *IDH2*, which are subunits of the mitochondrial NAD(+)-dependent isocitrate dehydrogenase that catalyzes the oxidation of isocitrate to α-ketoglutarate in the TCA cycle, and *IRC14*. *IRC14* is a dubious gene that overlaps with *IDH2*; thus high functional similarity to *IDH1* and *IDH2* is likely to be due to this overlap [41] (S1 Text). *IDH1* and *IDH2* are known to be regulated by Rtg1p and Rtg3p when the RTG pathway is activated [33]. The two subclusters that make up the sensor cluster thus capture a well-established signaling relationship between the RTG and TCA cycle.

PRS identified two histone genes *HHT1* (histone H3) and *HHF1* (histone H4), and an uncharacterized gene YDL159W-A, along the strongest PRS path connecting this sensor cluster with EC1 which is related to chromosome segregation. Interestingly, Ng et al. showed that several residues on histones H3 and H4 are critical for accurate chromosome segregation, causing defects in kinetochore stability and increased chromosome loss when mutated [42]. Based on the GI PSN network topology alone, the PRS path pinpointed this previously described relationship between chromosome segregation and the H3 and H4 histone genes, and predicted that H3 and H4 are involved in a critical indirect relationship with the TCA cycle / mitochondria-signaling. The molecular mechanisms linking TCA cycle / mitochondria-signaling to histones and chromosome segregation are not presently well characterized. However, Galdieri et al. showed that a decrease in the expression of the histones H3 and H4 can activate the RTG pathway, and increase the expression of *IDH1* and *IDH2* [43]. Therefore, taking all components of this PRS path together, a novel prediction emerges according to which the regulation of H3 and H4 expression levels could be a key mechanism linking the correct progression of chromosome segregation to central TCA metabolism through the RTG pathway. Based on our examination of this PRS path, PRS has the potential to capture biologically meaningful indirect relationships between cellular processes unbiasedly.

### Sensor and effector clusters are conserved properties of genetic networks

We showed that the existence of sensor clusters was a property of the biological structure of the yeast GI PSN as we were not able to observe sensor clusters in the randomized networks with the same degree distribution as the yeast GI PSN (S2 Fig). To investigate whether this is unique to the yeast GI PSN, we applied PRS to a GI PSN network in *Schizosaccharomyces pombe* [30] and a coessentiality network in human [44]. *S*. *pombe* GI PSN network was constructed similarly to the yeast GI PSN but is much smaller, based on the genetic interaction profile similarities between only 1,145 genes. On the other hand, the human coessentiality network contains 3,238 genes, and the edges represent single mutant profile similarities in 276 cancer lines as opposed to double mutant phenotypes. We applied the PRS strategy to identify sensors and effectors in these networks and investigated their topological and biological properties.

**Fig 6:**
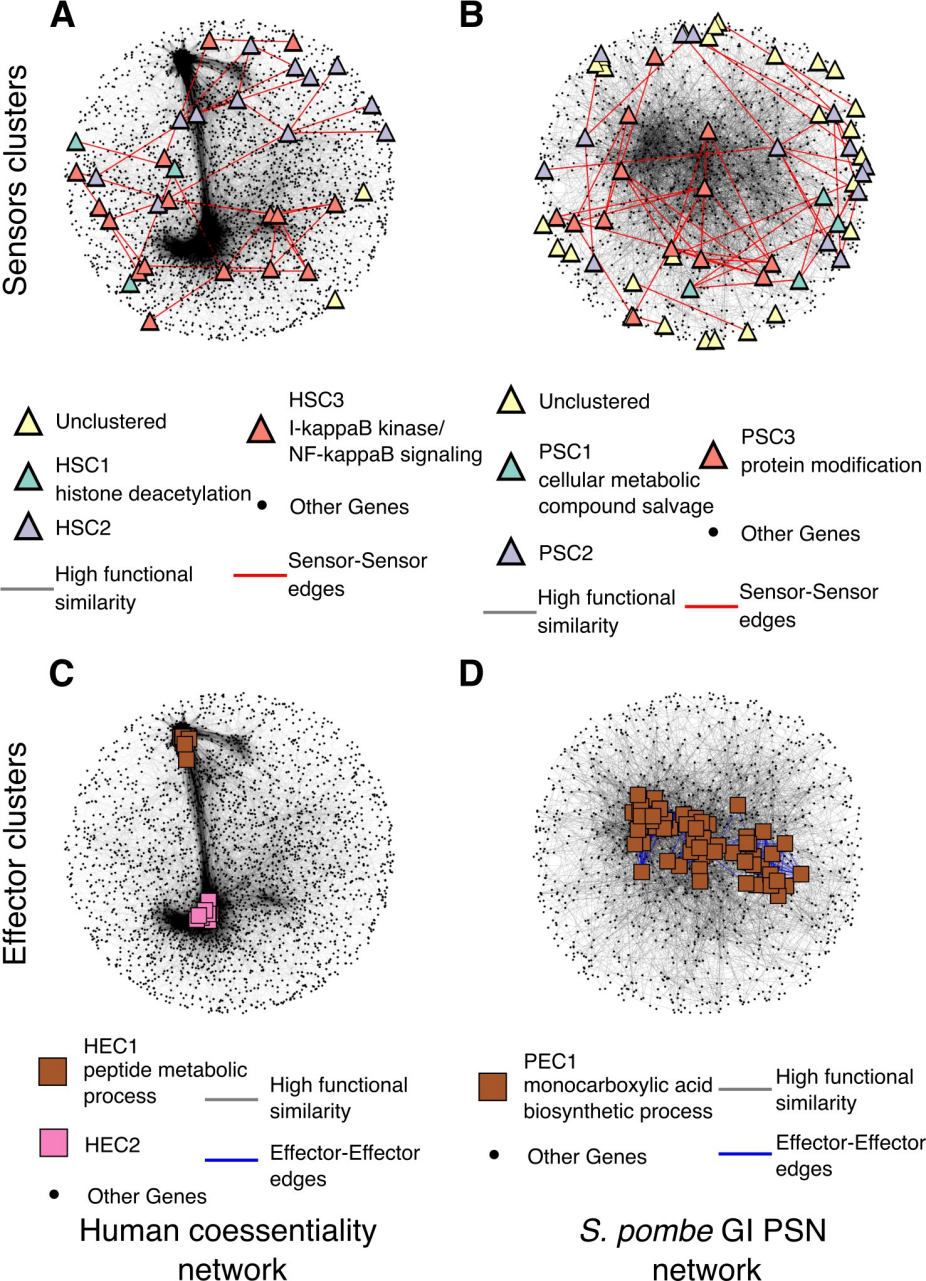
Genetic similarity networks for human and *Schizosaccharomyces pombe* show clusters of sensors and effectors. Sensors (*triangles*) and effectors (*squares*) in human coessentiality network (A-C, respectively) and in *S. pombe* GI PSN (B-D, respectively). Node colors represent sensor clusters (HSC1-3 in A, and PSC1-3 in B) or effector clusters (HEC1-2 in C or PEC1 in D). GO enrichments are indicated within SC or EC labels in the legends.

In line with our observations for the yeast GI PSN (Fig 2), we observed stronger effectiveness and sensitivity values for the human and *S*. *pombe* networks than for random networks of matched size and degree distribution, indicating that the real biological networks have a stronger propensity for indirect relationships than expected by chance (S6 and S7 Figs). We identified 32 sensors in the human coessentiality network (Fig 6A) and 57 sensors in the *S*. *pombe* GI PSN (Fig 6B), consisting of lower-degree genes (S6 and S7 Figs) and forming three sensor clusters in each network. Two sensor clusters in each network were enriched for GO biological process terms (Fig 6A and 6B, **S3** and **S4 Data**). Sensor clusters in the human coessentiality network were found to be enriched for IκB kinase/ NF-κB signaling or histone deacetylation (HSC1 and HSC3, resampling-based FDR = 0.002 and 0.038, respectively). Sensor clusters in the *S*. *pombe* GI PSN had GO enrichments for cellular metabolic compound salvage or protein modification terms (PSC1 and PSC3, resampling-based FDR = 0.075 and 0.036, respectively). The organization of sensor genes into network clusters with shared GO terms was not expected from the size and degree distribution of the human and *S*. *pombe* networks (S8 Fig, *p* < 0.01, empirical *p*-value, for both networks).

Both networks showed effector clusters consisting of higher degree genes (Figs 6C, 6D, S6 and S7). One of the effector clusters in the human coessentiality network was enriched for peptide metabolic processes and similar terms (HEC1, resampling-based FDR = 0.002, **S3 Data**) and the effector cluster in the *S*. *pombe* GI PSN was enriched for monocarboxylic acid metabolic processes (PEC1, resampling-based FDR = 0.048, **S4 Data**). Overall, our results reveal that the existence of central effector clusters and peripheral sensor clusters may be a fundamental property of genetic networks that is conserved across deep evolutionary distances.

## Discussion

In this study we adapted the PRS methodology, initially designed for characterizing allosteric signal transductions in molecular structures [20–22], to study the indirect relationships between gene (clusters) which are uniquely favored by the global architecture of genetic similarity networks. Our PRS analyses uncovered biologically coherent network clusters of effector and sensor genes critical for indirect relationships in yeast, human and *S*. *pombe*. Effectors are central to the network, whereas sensors tend to form antenna-shaped motifs at the network periphery. While the topological and biological importance of effectors could have been highlighted using other network analysis methods, we view the identification of critical sensor nodes as a key methodological innovation in the study of cellular networks. Sensors could not have been identified by existing propagation-based methods for the study of indirect relationships, which lack the ability to rank nodes based on their absolute propensity to integrate information, or by topology-based approaches such as centrality metrics or clustering techniques, which would forgo the sensors due to their low connectivity.

The perturbation effects that are generated by PRS correspond to the propagation of functional couplings through a network representation of the cell and cannot be directly equated with biological signaling in the molecular sense. Yet, we find that many (but not all) sensor genes identified by PRS in the yeast GI PSN are known to be involved in molecular signal transduction and cellular communication and that one sensor cluster (SC6) and one effector cluster (EC1) map to well-established signaling hubs. This suggests that the indirect relationships identified using PRS in the yeast GI PSN hold clues to generate novel hypotheses regarding the molecular mechanisms of cross-pathway signaling. We examined the genes mediating a key indirect relationship identified by PRS between an effector cluster and a sensor cluster. This led to the formulation of a novel hypothesis implicating a regulation of histone H3 and H4 expression levels as the possible key mechanism coordinating the correct progression of chromosome segregation to central carbon metabolism through induction of the mitochondria-nucleus retrograde pathway. Our work illustrates how the unbiased detection of indirect relationships between seemingly unrelated cellular processes can enable the generation of novel mechanistic hypotheses. We anticipate that many hypotheses emerging from PRS analyses of genetic similarity networks will fuel future studies and illuminate the molecular mechanisms of indirect relationships between cellular pathways and processes.

PRS analyses also provided novel insights about the high-level organization of indirect relationships in the cell. We found that the yeast, human and *S*. *pombe* genetic similarity networks all display a remarkable potential for indirect relationships relative to expectations based on their sizes and degree distributions. The organization of sensor nodes into clusters also distinguished the genetic networks from expectations. This suggests that evolutionary optimization for efficient molecular communication between pathways may translate to a network topology with enhanced capabilities for information sensing and transmitting. Such topological optimization may include the hierarchical organization of increasingly more connected clusters previously described for genetic similarity networks [27,30,44] as well as the lower-degree peripheral sensor clusters we discovered.

Altogether, our work demonstrates that PRS is a promising strategy for the unbiased study of indirect relationships in cellular networks. Beyond guilt-by-association [10] and local network context analyses [1], PRS provides an avenue to illuminate how genes can communicate and affect processes beyond their local neighborhood. Importantly, PRS eliminates the need to restrict these questions to source genes chosen based on prior knowledge. Our analyses add to the evidence [25,26] that spring-based physical modeling of cellular networks can be a powerful tool to uncover functional (effector or sensor) modules and their coupling within the higher-order organization of the cell. We hope that more insights will arise from future work modeling cellular networks as physical 3D objects.

We showed the applicability and utility of PRS analysis on three genetic similarity networks from different species and in theory, PRS can be applied to any network representation as long as Laplacian-based approaches are suitable to capture the graph structure [45]. Extremely large networks where the number of nodes approaches infinity may not be suitable. PRS may also be of limited use if the propagations are dominated by one or a few nodes in the network. This so-called “tip effect”, where large fluctuations of the free ends of a structure dominate the dynamics, may obscure the detection of more meaningful relationships withing the structure [46]. Despite these limitations, we anticipate that PRS strategies will become a useful tool for the study of complex networks in other realms of biology (e.g., protein interactions, gene regulation, etc.) and science (e.g., social, economic, etc.), where the identification of effectors and sensors of signals, or information, together with the PRS paths may reveal important communication hubs and lines.

## Materials and methods

### Yeast genetic interaction profile similarity network

We obtained the data from TheCellMap [41] (https://thecellmap.org/costanzo2016/, file: Genetic interaction profile similarity matrices). Details of the network construction can be found in the Supplementary Materials of Costanzo et al. [27] under the “Constructing genetic interaction profile similarity networks” section. In brief, the genetic interaction profile similarity between gene *i* and gene *j* is the Pearson’s correlation coefficient (PCC) between the genetic interaction profile vectors of *i* and *j*, which consist of genetic interaction scores experimentally estimated for all possible double mutants involving gene *i* or gene *j*:

$${Profilesimilarity}_{ij}=PCC(Profile_{i},Profile_{j}).$$

We used a PCC cutoff of 0.2 following the original publication [27], and constructed the GI PSN containing every gene with at least one profile similarity of PCC > 0.2. This resulted in a network with 5,272 nodes and 39,866 unweighted and undirected edges. To test the effect of chosen threshold we analyzed networks generated with PCC cutoffs of 0.05, 0.1, 0.25, 0.3, 0.35, and 0.4 and we presented the results in S1, S3 and S4 Figs. The visual network representations throughout the paper were generated using Fruchterman-Reingold force-directed layout [47] algorithm implemented in Python network analysis package *networkx* [48].

### Elastic network models and perturbation response scanning

We used the Gaussian network model (GNM) to represent the GI PSN as an elastic bead-and-spring network object. The overall connectivity of the network is represented by a Laplacian (also called Kirchhoff) matrix, whose diagonal elements are the degree of each node, and the non-zero, off-diagonal elements (equal to -1) indicate the connected pairs of nodes [23]. We first took the largest connected component of the GI PSN, which was represented by a GNM of *n* = 5,183 nodes and 39,816 edges. The corresponding Laplacian was used to perform the PRS analysis as described by Li et al. [49]. Mainly, we used *calcPerturbResponse* function in *ProDy* [50], a Python API designed originally for analyzing protein dynamics, to calculate the PRS matrix. PRS was originally formulated under the framework of the anisotropic network model (ANM) [20,21] and extended to the GNM by Li et al. [49]. In brief, the linear response theory states that in the presence of an external force, the positional displacements of all *n* nodes in the system under the equilibrium condition are dictated by the force balance

f=HΔr, (1)

where ***f*** and Δ***r*** are the 3*n*-dimensional external force and displacement vectors, respectively; and **H** is the 3*n*×3*n* force constant matrix called Hessian whose (pseudo) inverse is the 3D covariance matrix of the equilibrium fluctuations/displacement of the network nodes. Therefore, the displacements of the nodes are evaluated for a given ***f***, provided that the Hessian is known, using

$$\Delta r=H^{-1}f,$$ (2)

Under isotropic conditions represented by the GNM, the external forces and displacements can be reduced to *n*-dimensional vectors, $\Delta \tilde{r}$ and $\tilde{f}$, the elements of which represent the sizes of the displacements and forces corresponding to the *n* nodes; and the Hessian is replaced by the *n*×*n* symmetric connectivity matrix, or the so-called Kirchhoff or Laplacian, **Γ**, multiplied by a uniform spring constant *γ*. The above equation is then rewritten as

$$\Delta \tilde{r}=(\frac{1}{\gamma })\Gamma^{-1}\tilde{f}.$$ (3)

Note that this equation can be written for the response $\Delta \tilde{r}{|}_{i}$ of all residues to a force exerted on the node *i*, i.e.,

$$\Delta \tilde{r}{|}_{i}=(\frac{1}{\gamma })\Gamma^{-1}{\tilde{f}}_{i},$$ (4)

where ${\tilde{f}}_{i}={\left[0\;0\ldots 0\;{\tilde{f}}_{i}\;0\ldots 0\right]}^{T}$ is the force vector exerted on node *i*, all elements of which are zero, except the *i*^*th*^, ${\tilde{f}}_{i}$. The PRS matrix **P** in the GNM is evaluated by repeating the above operation for all nodes 1≤*i*≤*n*, and normalization of the result to remove the intrinsic biases (node-specific variance *σ*_*i*_), i.e.,

$$P\equiv [\Delta \tilde{r}{|}_{1}\Delta \tilde{r}{|}_{2}\ldots .\Delta \tilde{r}{|}_{n}]=(\frac{1}{\gamma })\Gamma^{-1}{\left[diag(\sigma_{1},\sigma_{2}.\ldots .\sigma_{n})\right]}^{-1}[{\tilde{f}}_{1}{\tilde{f}}_{2}\ldots {\tilde{f}}_{n}]$$ (5)

where $\left[{\tilde{f}}_{1}\;{\tilde{f}}_{2}\ldots {\tilde{f}}_{n}\right]$ is an *n*×*n* matrix the *i*^*th*^ column of which is ${\tilde{f}}_{i},\;{(1/\gamma )\Gamma }^{-1}$ serves as the transfer function, and the diagonal matrix [diag(*σ*_1_, *σ*_2_.….*σ*_*n*_)]^−1^ is the normalization factor. The *ij*^*th*^ element *P*_*ij*_ of **P** represents the response of node *j* to perturbation at node *i*. We note that *σ*_*i*_ simply represents the square-fluctuation of node *i* under equilibrium conditions, which is an intrinsic property uniquely defined by the network topology and readily computed by the GNM as the *i*^*th*^ diagonal element of the covariance matrix (which is inversely proportional to **Γ**). The normalization accounts for the topology-defined adaptability of the nodes and breaks the symmetry of the covariance matrix. The row and column averages of the PRS matrix give the effectiveness and the sensitivity profiles as a function of gene index [1, *n*], respectively.

### *S. pombe* genetic interaction profile similarity network

We obtained data from Ryan et al. [30] (file: supplementary data S3). Ryan et al. used PCC between genetic interaction profiles and then converted PCC values into a similarity metric using logistic regression (see Ryan et al. supplementary material, *Similarity score* section). We used similarity score threshold of 0.1 to construct the network, as it was suggested in Ryan et al. This generated a network with 1,272 nodes and 5,011 unweighted edges. The giant component of this network with 1,145 nodes and 4,933 edges was used for PRS analyses as explained above. Genes with the top 5% of the effectiveness and sensitivity values were taken as effectors and sensors for this network.

### Human coessentiality network

We obtained data from Kim et al. [44] (file: supplementary table S5). This dataset represents high confidence positive profile similarities with Bonferroni-corrected *p*-values less than 0.05. The network contained 3,483 nodes and 68,813 edges with the giant component having 3,238 nodes and 68,641 edges. The giant component of this network was used for PRS analyses as explained above. Genes with the top 1% of the effectiveness and sensitivity values were taken as effectors and sensors for this network.

### PRS matrix clustering

To cluster the PRS matrix elements, we used a hierarchical clustering algorithm implemented in the Python package *SciPy*. We first capped the outliers in the PRS matrix by normalizing the values above 95% of the matrix to be equal to 95% value. Then we calculated the pairwise standardized Euclidean distance between genes using rows or columns of the PRS matrix as the coordinates and used *ward* linkage metric to construct a dendrogram of the genes. The distinct perturbed and responding gene clusters were extracted by cutting the dendrogram tree to create two sub-dendrograms and taking the smaller sub-dendrogram as the distinct perturbed or responding gene clusters. Clustered heatmap and dendrograms were visualized using R package *ComplexHeatmap* [51].

### Permutation test

We calculated the significance of differences between distinct perturbed and responding gene clusters and other genes in the networks using the following permutation test. We shuffled the effectiveness (or sensitivity) values of all genes and calculated the differences between mean values of distinct perturbed cluster (or responding cluster) and other genes. We then used empirical *p*-value formula following Davison and Hinkley [52]:

$$p=\frac{r+1}{n+1}$$ (6)

where *r* is how many times the unshuffled difference in means is smaller than shuffled difference in means and *n* is the total number of shuffling (*n* = 10000, in this calculation). The significance level was taken as *α*<0.05.

### Network properties

The following definitions are used. Node degree is the number of edges of a given node. Average neighbor degree is the average degree of the first neighboring nodes of a given node. Percentage of in-between edges for a given group of nodes is the ratio of the total number of edges that are directly connecting the nodes in the group to the total number of edges the nodes in the group have.

The relationships of effectiveness and sensitivity with the degree distribution were calculated by using two measures of correlations: Pearson’s correlation coefficient (*R*) and Spearman’s rank correlation coefficient (*ρ*). *R* was used to measure effectiveness and degree correlation. *ρ* was used to measure sensitivity and degree correlation. *cor*.*test* function in R was used to measure these values by changing the *method* parameter, respectively.

### Network rewiring

To rewire the network while keeping the degree distribution the same, we applied an edge swapping procedure. A swap between two randomly selected edges is accepted if the network connectivity is not violated, i.e., no network node is disconnected from the network, and if the newly generated edges are not already in the network. This process is repeated a minimum of 10 times the number of edges in the network. The resulting rewired network maintains the same degree for each node as the original network but has different connections. For this process, we used *connected_double_edge_swap* function of *networkx*.

### Empirical p-value calculations

Empirical *p*-values were calculated using rewired network properties. For each property we sought to investigate its significance, we compared the mean value calculated using the real GI PSN network to mean values calculated from 100 rewired networks. We then used the same empirical *p*-value formula in Eq 6 where *r* is the number of times the mean of the real network property is smaller (or bigger, depending on the hypothesis) than the mean of the rewired network properties and *n* is the total number of rewired networks (*n* = 100, in this analysis).

### Gene ontology enrichment analyses

GO trees (file: *go-basic*.*obo*) and annotations (files: *sgd*.*gaf*, *pombase*.*gaf*, *goa_human*.*gaf*) were downloaded from http://geneontology.org/ on March 10, 2022. We used the Python package, *GOATools* [53], to calculate the number of genes associated with each GO term in the study group and the overall population of (all) genes for each respective network. We excluded annotations based on the evidence codes ND (no biological data available), IGI (inferred from genetic interaction), and HGI (inferred from high throughput genetic interaction) to remove any associations originating from the networks we used. We identified GO term enrichments by calculating the likelihood of the ratio of the genes associated with a GO term within a study group (e.g., a sensor or an effector cluster) given the total number of genes associated with the same GO term in the background set of all genes in the network. We applied Fisher’s exact test and resampling-based false discovery rate (FDR) [54] multiple testing correction to calculate corrected *p*-values for the enrichment of GO term in the study group. For each GO enrichment test, we calculated *p*-values for a random set of same size for 500 times to generate an empirical *p*-value distribution. FDR is then calculated using Eq 6 where r is the number of cases that real *p*-values being smaller than empirical *p*-values. FDR < 0.1 was taken as a requirement for significance.

### Sensors and effectors group comparisons

Kruskal-Wallis test was used to statistically investigate the differences between effector or sensor groups in terms of their degree, effectiveness or sensitivity values for the analyses shown in Figs 3D and 3E, 4C and 4D. We applied *kruskal*.*test* function in R with a significance level of *α* = 0.05. To find the group that deviates from the null model, we used Tukey’s HSD test [55], which is equivalent to a pairwise Wilcoxon test with multiple testing corrections.

### PRS path analysis

For each path starting at gene *i*, we took *i*^*th*^ row values, which represents the response of every gene when gene *i* is perturbed, of the PRS matrix as node weights. To find the shortest path with the maximum information, we identified unweighted shortest paths from node *i* to all other nodes. When there are multiple shortest paths, we selected the one with the maximum sum of node weights. To identify a PRS path between clusters of genes, we applied the same strategy to all pairs of genes in two clusters and selected the shortest path with highest weight as shortest path between clusters. Note that for the cases where there is only single shortest path, PRS path and shortest path are the same. Cytoscape [25] and *networkx* were used to visualize the paths between effectors and sensors. GO enrichment analysis was done as explained above using the genes that are on the paths between effector and sensor clusters.

## Supporting information

S1 Data Significant GO terms with FDR < 0.1 for sensor and effector clusters in the yeast GI PSN. (CSV)

S2 Data 15 sensor genes in the yeast GI PSN with cellular roles in signaling. (CSV)

S3 Data Significant GO terms with FDR < 0.1 for sensor and effector clusters in the human coessentiality network. (CSV)

S4 Data Significant GO terms with FDR < 0.1 for sensor and effector clusters in the *S*. *pombe* GI PSN. (CSV)

S1 Text Overlapping genes and neighboring gene effects. (DOCX)

S1 Fig Real networks show higher information propagation potential irrespective of chosen Pearson’s correlation coefficient between genetic interaction profiles (PCC) threshold. Difference of effectiveness (A) and sensitivity (B) profiles between real (*solid cyan*) and randomly rewired networks (*dashed red*) for different PCC thresholds.
(TIF)

S2 Fig Rewired networks show significantly less sensor clusters compared to real network. A) Number of sensor clusters found for rewired networks compared to the real GI PSN. B) Number of GO enriched sensor clusters found for rewired networks compared to the real GI PSN. C) Number of antenna motifs out of 68 rewired networks which had sensor clusters. D) An example rewired network with the same degree distribution of the GI PSN, map showing sensors. It can be seen that while there are same number of sensors (n = 52) identified, there are only a handful of sensor-sensor edges, meaning no sensor clusters are formed, as opposed to clusters formed in the real PSN.
(TIF)

S3 Fig Different PCC thresholds result in distinct sensor characteristics with similar antenna motifs. A) Percentage of common sensors found when using different PCC thresholds (percentage of the common sensors between the thresholds shown on the x and y axes divided by the number of sensors identified at threshold shown on y-axis). There are several reasons we observe these differences. Due to the mathematical nature of GI PSN, the change of PCC thresholds will lead to different networks. For example, networks constructed with higher thresholds contain genes with higher similarity. Costanzo et al. [27] showed that these different networks represent different biology. Their results showed that a PCC ≥ 0.05 would be enriched in co-localization relationships while a PCC ≥ 0.4 would be enriched in pathways and protein complexes. Additionally, higher thresholds would result in a smaller network. Taken together, the identification of different sensors in different GI PSN networks is not surprising. B) Number of sensor clusters identified at the given threshold. At PCC ≥ 0.2 we identify the most sensors. When PCC < 0.2 thresholds are used, the network is near complete, thus sensors create a single component that cannot be separated into different connected components. For PCC ≥ 0.25, the increased threshold leads to smaller networks, thus fewer sensors, and sensor clusters. C) Number of GO enriched sensor clusters at different thresholds. D) Sensors in different PCC threshold networks. Similar to Fig 3F, most sensor clusters show antenna motifs where the sensors are connected to the rest of the network via a single node.
(TIF)

S4 Fig Effectors in different threshold networks show similarities. Percentage of common effectors (A) and common GO terms found for effectors (B) when using different PCC thresholds (overlap for the thresholds shown on x and y axes divided by the total number when using the threshold on y-axis). PCC threshold 0.2, which we used as default for our study, has many common effectors found in higher threshold networks. The higher overlap on the identified effectors and GO terms could be expected given the degree of the effectors. Due to the higher degree of the effectors in the PCC ≥ 0.2 network, they are less likely to be removed from the network and keep their relatively higher degree in higher threshold networks.
(TIF)

S5 Fig PRS can identify the most information carrying paths among thousands of possible shortest paths between effectors and sensors. A) Each cell shows the number of shortest paths identified between the effector cluster shown on the y-axis and sensor cluster shown on the x-axis when considering all possible shortest paths between all effector-sensor pairs for each cluster. B-D) All shortest paths between three effector clusters and nine sensor clusters which are chosen based on their high PRS signal which is calculated by the sum of response magnitudes of genes on a shortest path.
(TIF)

S6 Fig The human coessentiality network displays superior propagation potential compared to randomly rewired networks with identical degree distributions. A) Degree and effectiveness scatter plot shows strong correlation between degree and effectiveness in the human coessentiality network (*R* = 0.99). B) Degree and sensitivity scatter plot shows a small positive correlation between degree and sensitivity in the human coessentiality network (*ρ* = 0.14). C) Degree distributions for the human coessentiality network (*cyan*) and 100 rewired networks (*red*). These distributions overlap by design. D) The correlation between degree and effectiveness is significantly higher in the human coessentiality network (*cyan vertical line*) than that expected for the rewired networks (*dashed red distribution*, average *R* = 0.94, *p* < 0.01, empirical *p*-value). E) The correlation between degree and sensitivity is significantly weaker and has a different sign in the human coessentiality network (*cyan vertical line*) compared to expectations from rewired networks (*dashed red distribution*, average *ρ* = -0.88, *p* < 0.01, empirical *p*-value). Nodes in the human coessentiality network (*cyan distributions*) exhibit significantly higher effectiveness (F) and sensitivity (G) compared to random network nodes (*red dashed distributions*, *p* < 0.01, empirical *p*-value).
(TIF)

S7 Fig The *S*. *pombe* GI PSN coessentiality network displays superior propagation potential compared to randomly rewired networks with identical degree distributions. A) Degree and effectiveness scatter plot shows strong correlation between degree and effectiveness in *S*. *pombe* GI PSN (*R* = 0.88). B) Degree and sensitivity scatter plot shows a small negative correlation between degree and sensitivity in *S*. *pombe* GI PSN (*ρ* = -.29). C) Degree distributions for *S*. *pombe* GI PSN (*cyan*) and 100 rewired networks (*red*). These distributions overlap by design. D) The correlation between degree and effectiveness is significantly higher in *S*. *pombe* GI PSN (*cyan vertical line*) than that expected for the rewired networks (*dashed red distribution*, average *R* = 0.82, *p* < 0.01, empirical *p*-value). E) The correlation between degree and sensitivity is significantly weaker in *S*. *pombe* GI PSN (*cyan vertical line*) than expected from rewired networks (*dashed red distribution*, average *ρ* = -0.98, *p* < 0.01, empirical *p*-value). Nodes in *S*. *pombe* GI PSN (*cyan distributions*) exhibit significantly higher effectiveness (F) and sensitivity (G) compared to random network nodes (*red dashed distributions*, *p* < 0.01, empirical *p*-value).
(TIF)

S8 Fig Rewired networks show less sensor clusters compared to real networks. A) Number of sensor clusters found for rewired networks compared to the real human coessentiality network. B) Number of GO enriched sensor clusters found for rewired networks compared to the real human coessentiality network. C) Number of sensor clusters found for rewired networks compared to the real *S*. *pombe* GI PSN. B) Number of GO enriched sensor clusters found for rewired networks compared to the real *S*. *pombe* GI PSN.
(TIF)
