# Supplementary material for: Elastic network modeling of cellular networks unveils sensor and effector genes that control information flow
Source: PLoS Comput Biol. 2022 May 31;18(5):e1010181. doi: 10.1371/journal.pcbi.1010181 (PMC9216591; doi:10.1371/journal.pcbi.1010181)
Supplement: S1 Text — (DOCX) [file pcbi.1010181.s005.docx]

# Supplementary text

## Appendix 1

**Overlapping genes and neighboring gene effects**

In certain cases, the true cause of a genetic interaction might be difficult to pinpoint. For instance, deletion of overlapping or neighboring genes could cause partial deletion of the other gene, thus it is difficult to assign a genetic interaction to either gene. It has been previously shown that overlapping or neighboring genes share many genetic interactions and have high genetic interaction profile similarity[2]. Particularly, dubious open reading frames which do not have experimental data that show a gene product or lack conservation in Saccharomyces species tend to overlap with verified genes. We decided to keep overlapping and neighboring genes for two reasons. First, we wanted to follow the original publication [1] where there was no filtering based on overlap or proximity to other genes. Second, following Usaj et al. [2], while the overlapping genes might share a significant number of genetic interactions, they do not show 100% similarity. The shared genetic interactions could be regarded as the confirmation of the interactions of an actual characterized gene, but the unshared one could be due to deletion of dubious genes or could be interactions of characterized genes that were not detected in the original deletion. Thus, keeping these overlapping or neighboring genes we keep more information about the network.

# References

1. Costanzo M, VanderSluis B, Koch EN, Baryshnikova A, Pons C, Tan G, et al. A global genetic interaction network maps a wiring diagram of cellular function. Science. 2016;353. doi:10.1126/science.aaf1420

2. Usaj M, Tan Y, Wang W, VanderSluis B, Zou A, Myers CL, et al. TheCellMap.org: A Web-Accessible Database for Visualizing and Mining the Global Yeast Genetic Interaction Network. G3 (Bethesda). 2017;7: 1539–1549. doi:10.1534/g3.117.040220
