# Supplementary figures and images for: Elastic network modeling of cellular networks unveils sensor and effector genes that control information flow
Source: PLoS Comput Biol. 2022 May 31;18(5):e1010181. doi: 10.1371/journal.pcbi.1010181 (PMC9216591; doi:10.1371/journal.pcbi.1010181)

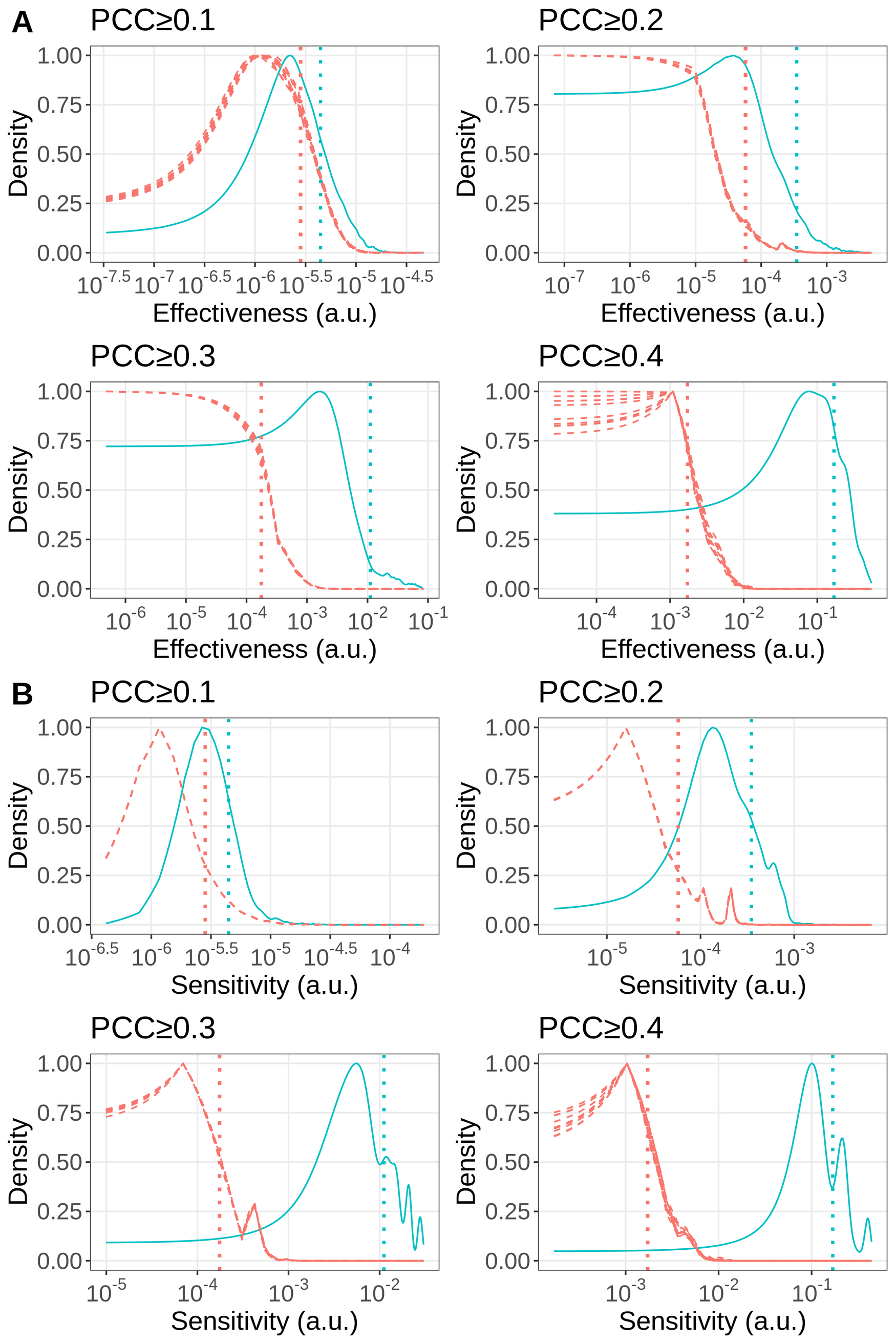

Supplement: S1 Fig — Difference of effectiveness (A) and sensitivity (B) profiles between real (solid cyan) and randomly rewired networks (dashed red) for different PCC thresholds. (TIF) [file pcbi.1010181.s006.tif]

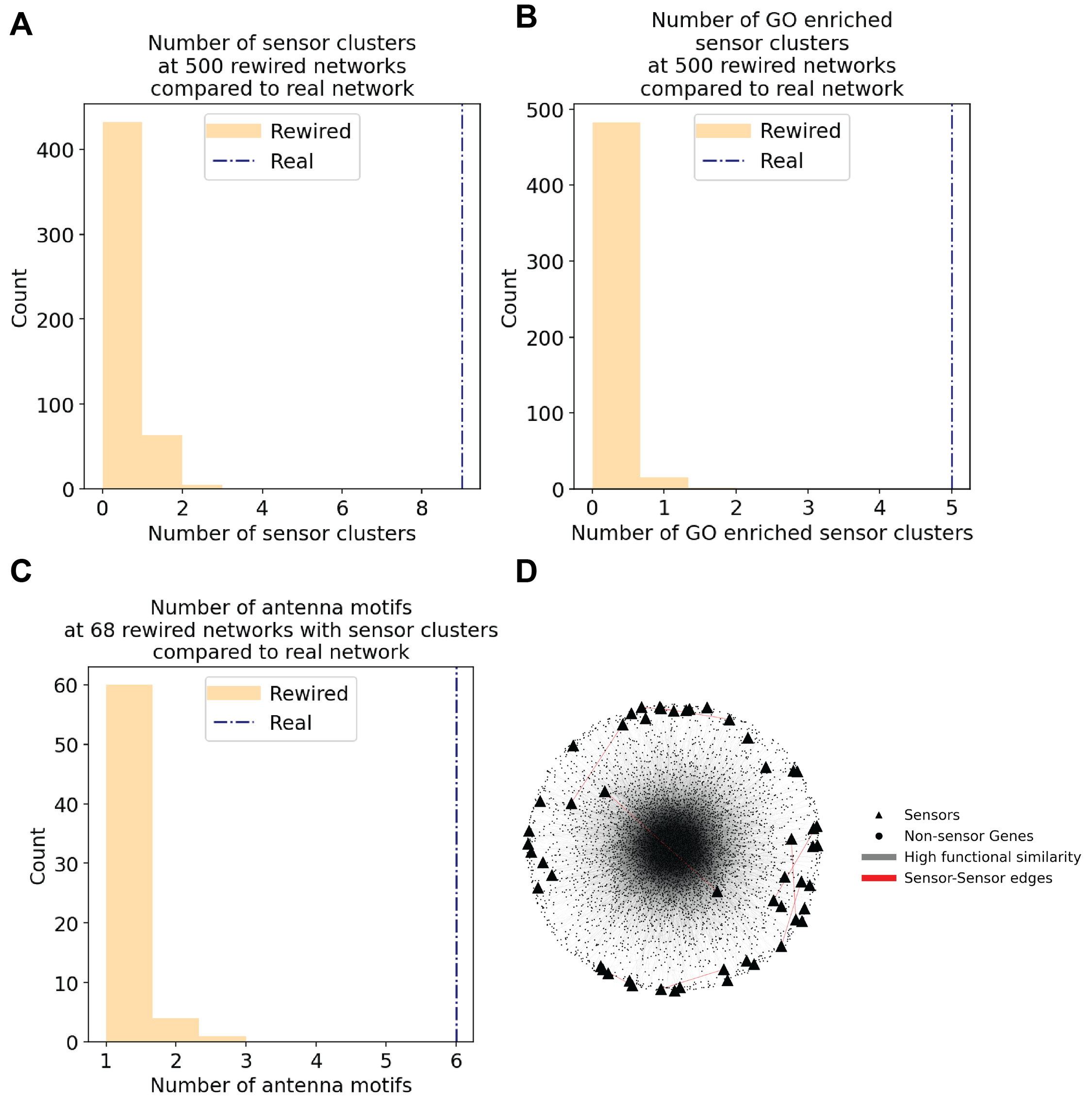

Supplement: S2 Fig — A) Number of sensor clusters found for rewired networks compared to the real GI PSN. B) Number of GO enriched sensor clusters found for rewired networks compared to the real GI PSN. C) Number of antenna motifs out of 68 rewired networks which had sensor clusters. D) An example rewired network with the same degree distribution of the GI PSN, map showing sensors. It can be seen that while there are same number of sensors (n = 52) identified, there are only a handful of sensor-sensor edges, meaning no sensor clusters are formed, as opposed to clusters formed in the real PSN. (TIF) [file pcbi.1010181.s007.tif]

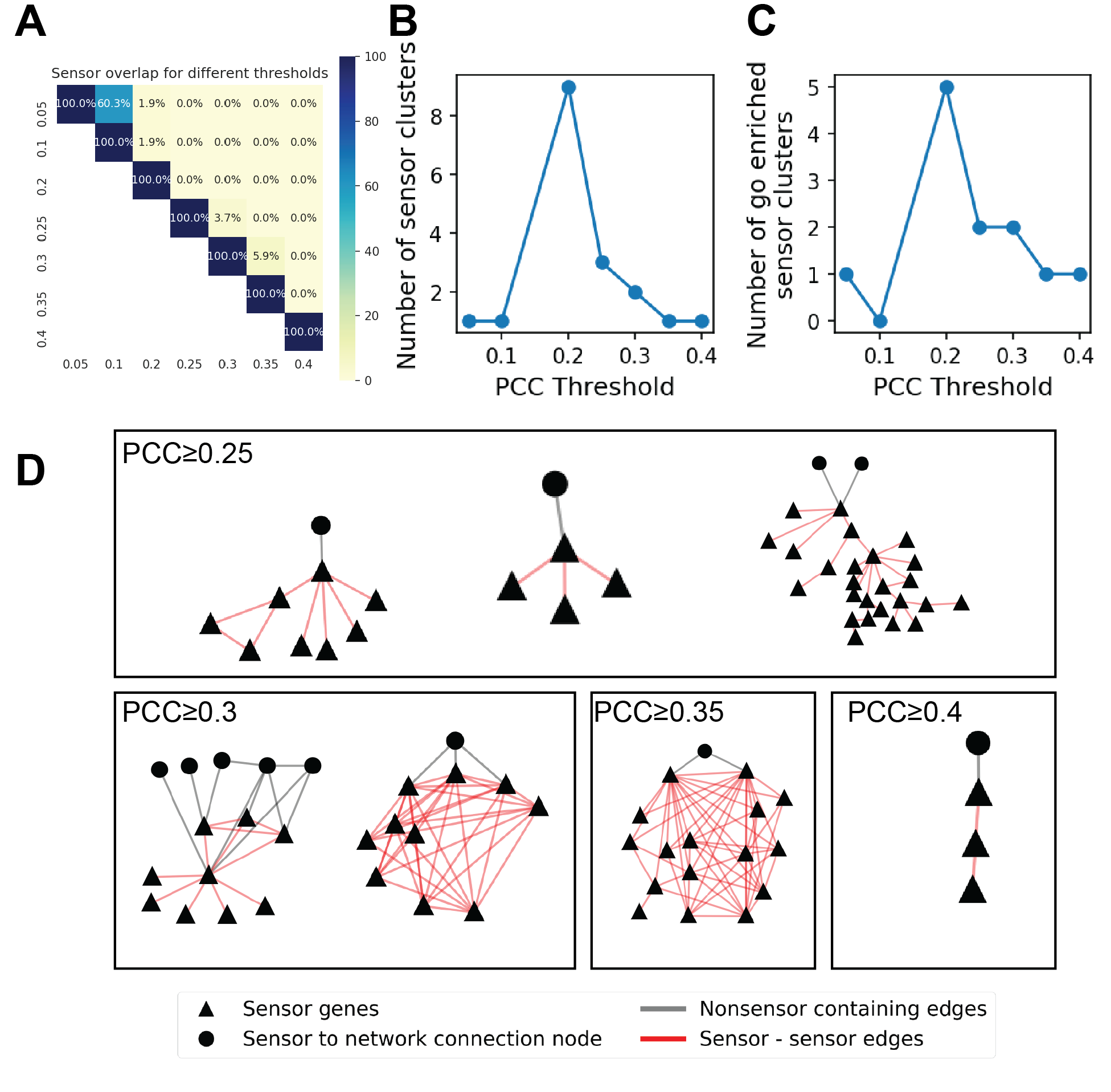

Supplement: S3 Fig — A) Percentage of common sensors found when using different PCC thresholds (percentage of the common sensors between the thresholds shown on the x and y axes divided by the number of sensors identified at threshold shown on y-axis). There are several reasons we observe these differences. Due to the mathematical nature of GI PSN, the change of PCC thresholds will lead to different networks. For example, networks constructed with higher thresholds contain genes with higher similarity. Costanzo et al. [27] showed that these different networks represent different biology. Their results showed that a PCC ≥ 0.05 would be enriched in co-localization relationships while a PCC ≥ 0.4 would be enriched in pathways and protein complexes. Additionally, higher thresholds would result in a smaller network. Taken together, the identification of different sensors in different GI PSN networks is not surprising. B) Number of sensor clusters identified at the given threshold. At PCC ≥ 0.2 we identify the most sensors. When PCC < 0.2 thresholds are used, the network is near complete, thus sensors create a single component that cannot be separated into different connected components. For PCC ≥ 0.25, the increased threshold leads to smaller networks, thus fewer sensors, and sensor clusters. C) Number of GO enriched sensor clusters at different thresholds. D) Sensors in different PCC threshold networks. Similar to Fig 3F, most sensor clusters show antenna motifs where the sensors are connected to the rest of the network via a single node. (TIF) [file pcbi.1010181.s008.tif]

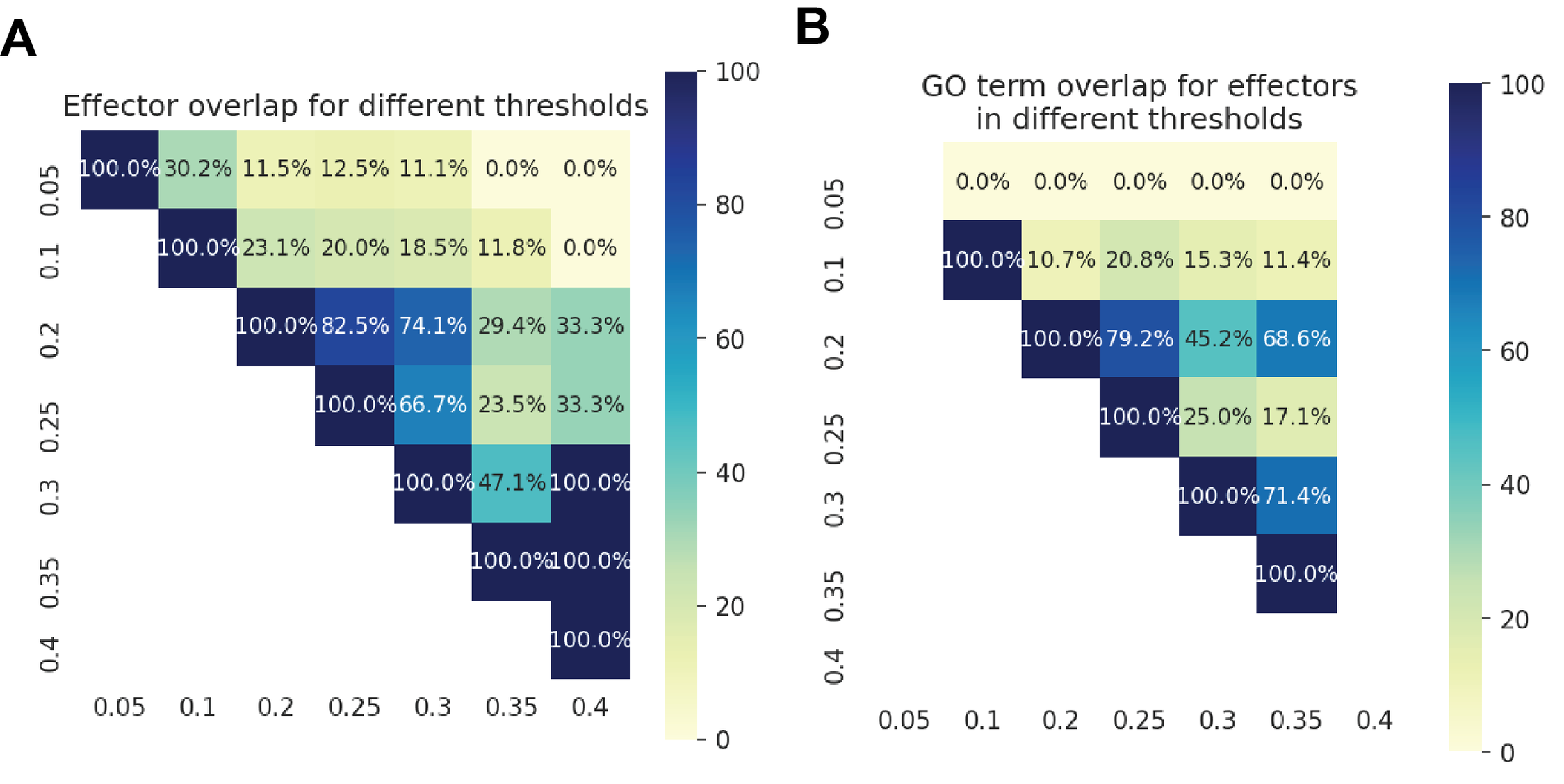

Supplement: S4 Fig — Percentage of common effectors (A) and common GO terms found for effectors (B) when using different PCC thresholds (overlap for the thresholds shown on x and y axes divided by the total number when using the threshold on y-axis). PCC threshold 0.2, which we used as default for our study, has many common effectors found in higher threshold networks. The higher overlap on the identified effectors and GO terms could be expected given the degree of the effectors. Due to the higher degree of the effectors in the PCC ≥ 0.2 network, they are less likely to be removed from the network and keep their relatively higher degree in higher threshold networks. (TIF) [file pcbi.1010181.s009.tif]

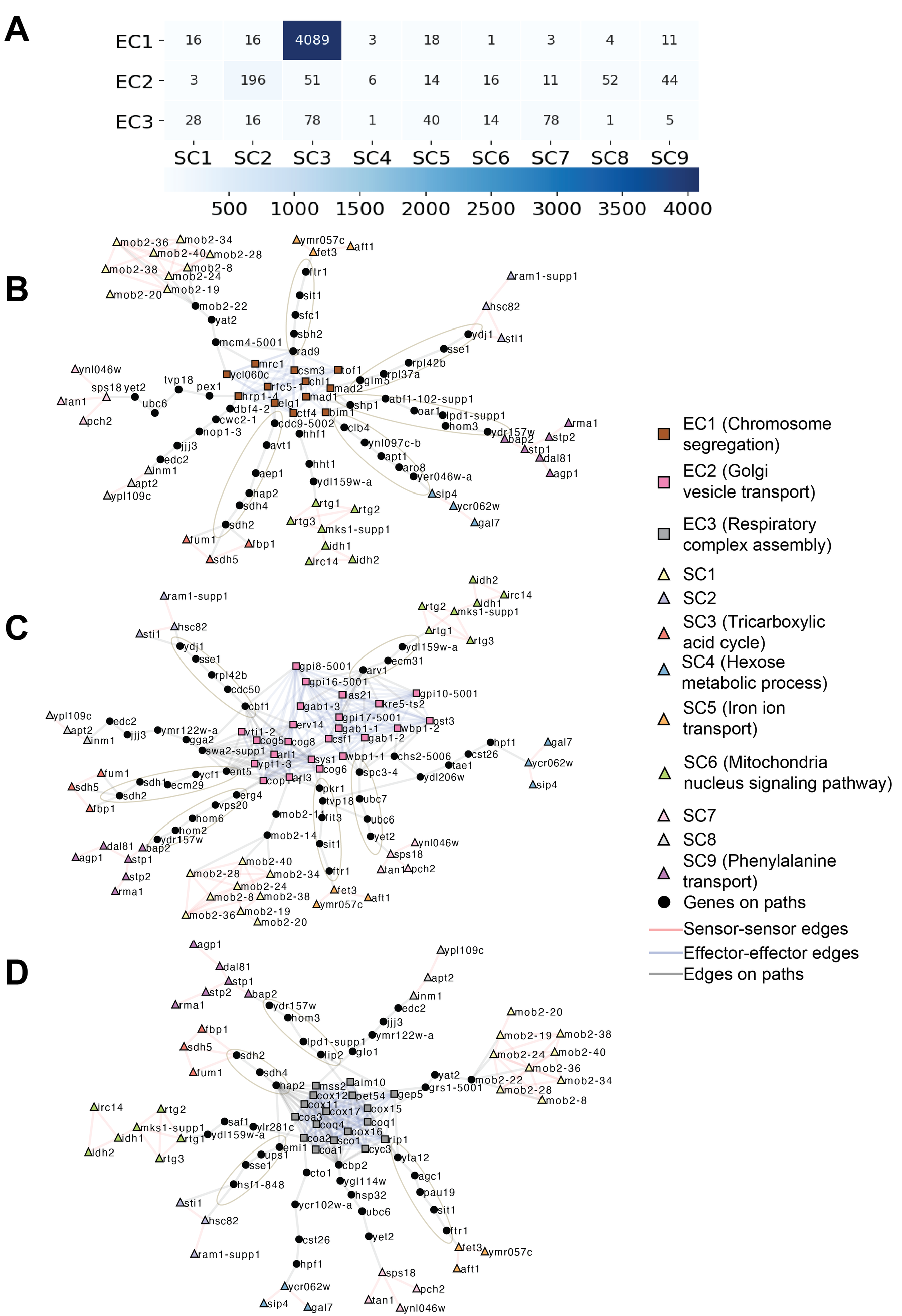

Supplement: S5 Fig — A) Each cell shows the number of shortest paths identified between the effector cluster shown on the y-axis and sensor cluster shown on the x-axis when considering all possible shortest paths between all effector-sensor pairs for each cluster. B-D) All shortest paths between three effector clusters and nine sensor clusters which are chosen based on their high PRS signal which is calculated by the sum of response magnitudes of genes on a shortest path. (TIF) [file pcbi.1010181.s010.tif]

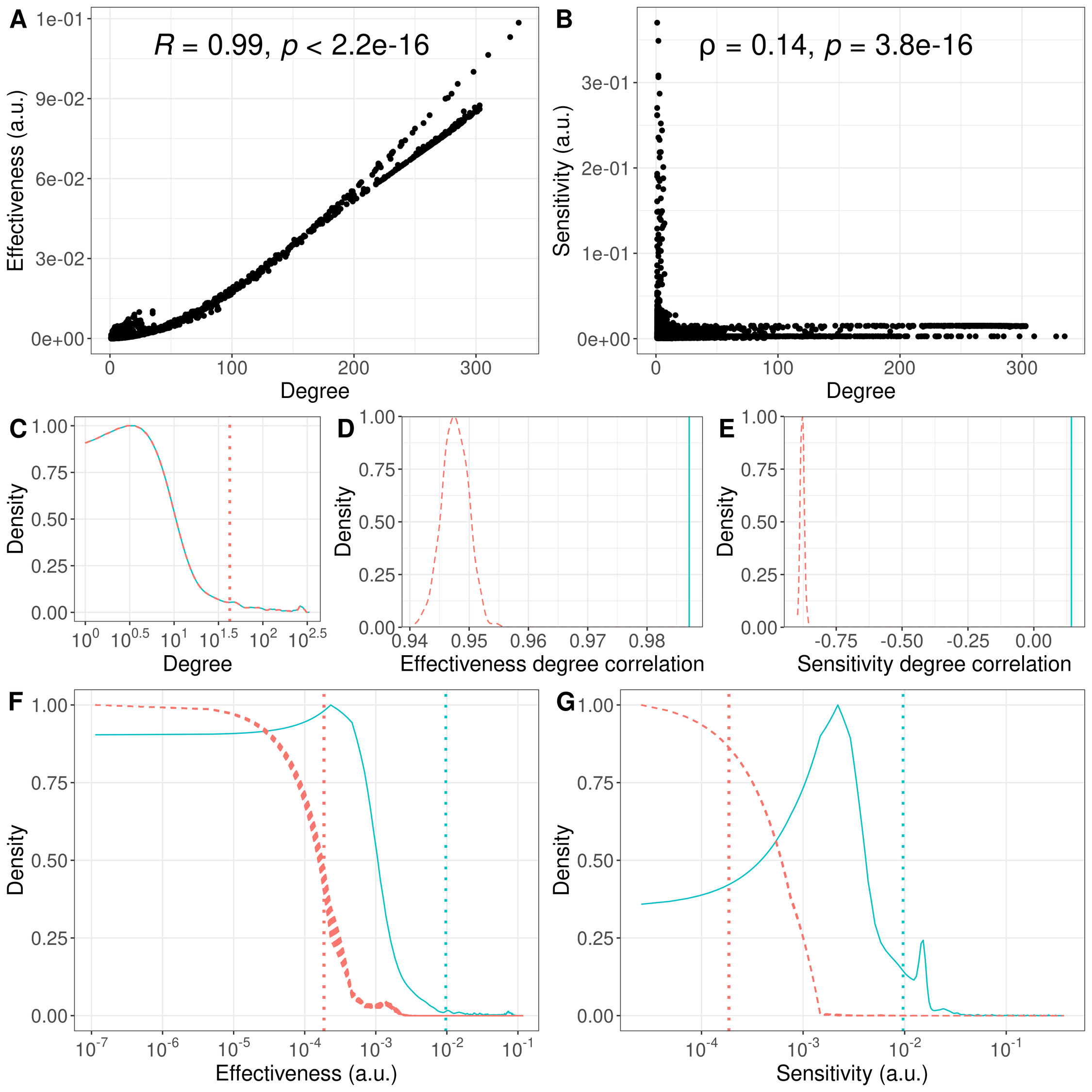

Supplement: S6 Fig — A) Degree and effectiveness scatter plot shows strong correlation between degree and effectiveness in the human coessentiality network (R = 0.99). B) Degree and sensitivity scatter plot shows a small positive correlation between degree and sensitivity in the human coessentiality network (ρ = 0.14). C) Degree distributions for the human coessentiality network (cyan) and 100 rewired networks (red). These distributions overlap by design. D) The correlation between degree and effectiveness is significantly higher in the human coessentiality network (cyan vertical line) than that expected for the rewired networks (dashed red distribution, average R = 0.94, p < 0.01, empirical p-value). E) The correlation between degree and sensitivity is significantly weaker and has a different sign in the human coessentiality network (cyan vertical line) compared to expectations from rewired networks (dashed red distribution, average ρ = -0.88, p < 0.01, empirical p-value). Nodes in the human coessentiality network (cyan distributions) exhibit significantly higher effectiveness (F) and sensitivity (G) compared to random network nodes (red dashed distributions, p < 0.01, empirical p-value). (TIF) [file pcbi.1010181.s011.tif]

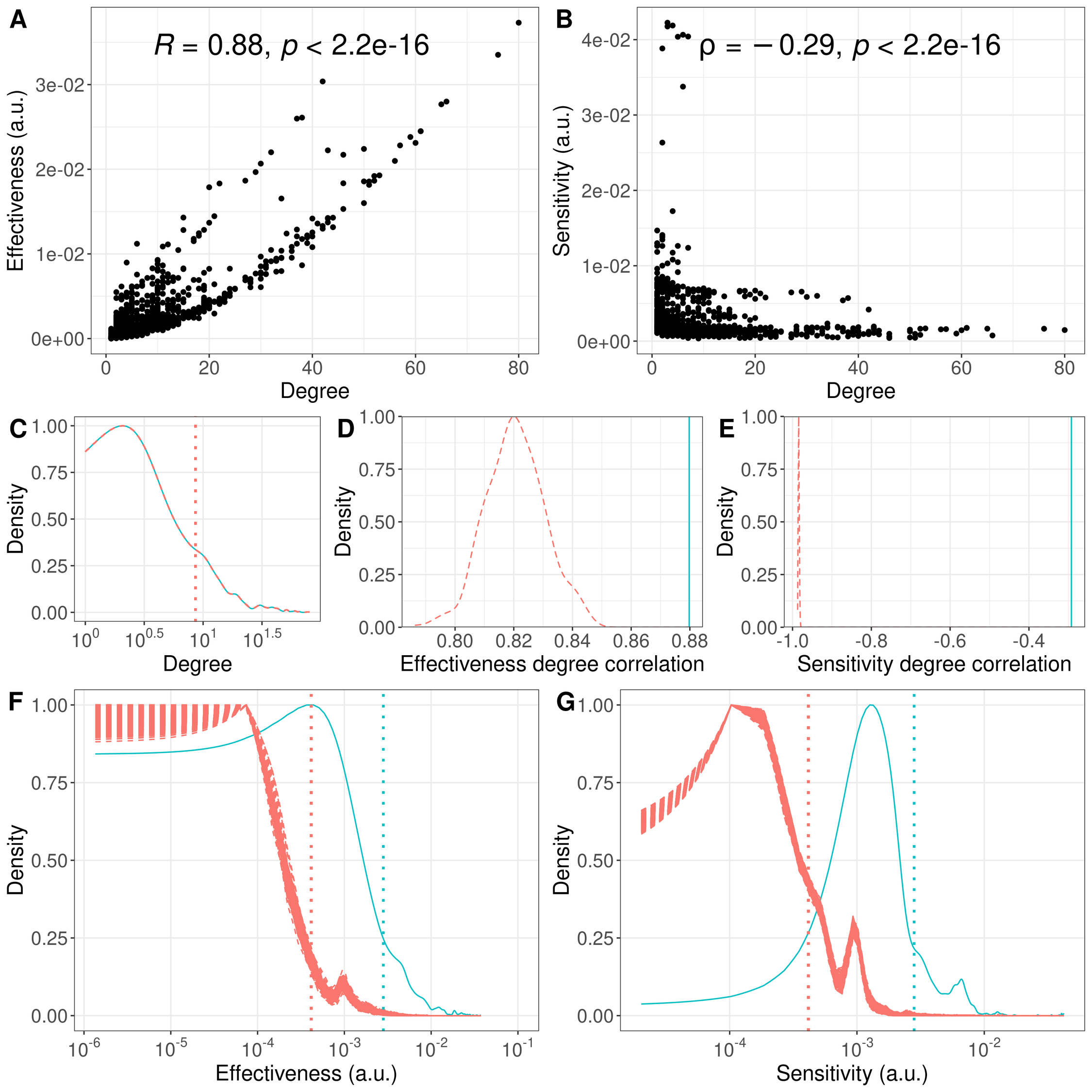

Supplement: S7 Fig — A) Degree and effectiveness scatter plot shows strong correlation between degree and effectiveness in S. pombe GI PSN (R = 0.88). B) Degree and sensitivity scatter plot shows a small negative correlation between degree and sensitivity in S. pombe GI PSN (ρ = -.29). C) Degree distributions for S. pombe GI PSN (cyan) and 100 rewired networks (red). These distributions overlap by design. D) The correlation between degree and effectiveness is significantly higher in S. pombe GI PSN (cyan vertical line) than that expected for the rewired networks (dashed red distribution, average R = 0.82, p < 0.01, empirical p-value). E) The correlation between degree and sensitivity is significantly weaker in S. pombe GI PSN (cyan vertical line) than expected from rewired networks (dashed red distribution, average ρ = -0.98, p < 0.01, empirical p-value). Nodes in S. pombe GI PSN (cyan distributions) exhibit significantly higher effectiveness (F) and sensitivity (G) compared to random network nodes (red dashed distributions, p < 0.01, empirical p-value). (TIF) [file pcbi.1010181.s012.tif]

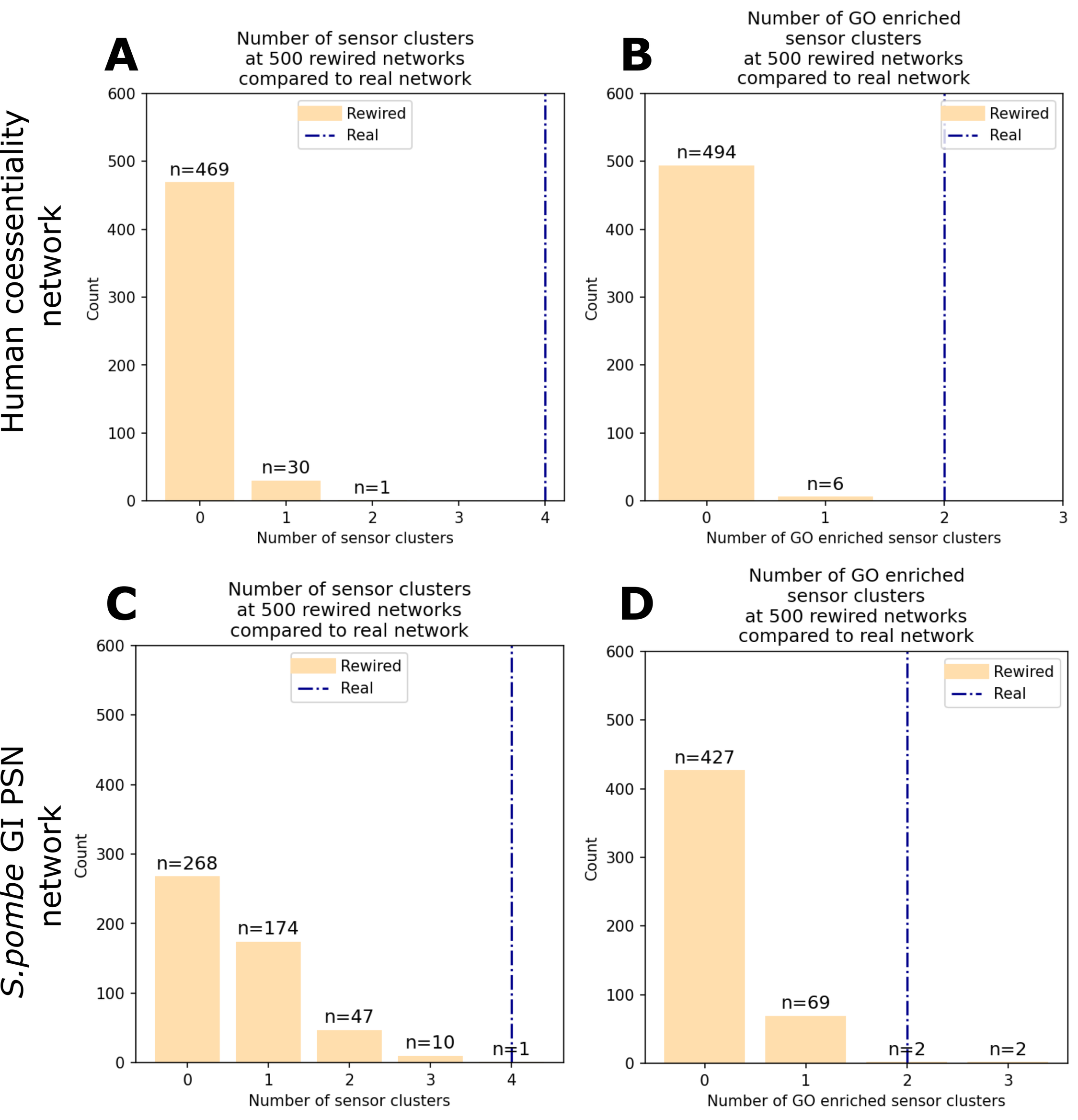

Supplement: S8 Fig — A) Number of sensor clusters found for rewired networks compared to the real human coessentiality network. B) Number of GO enriched sensor clusters found for rewired networks compared to the real human coessentiality network. C) Number of sensor clusters found for rewired networks compared to the real S. pombe GI PSN. B) Number of GO enriched sensor clusters found for rewired networks compared to the real S. pombe GI PSN. (TIF) [file pcbi.1010181.s013.tif]
